# Repairing Effect and Mechanism of the 4-Dimensionally Printed Limbal Stem Cell Strategy on Corneal Alkali Burns in Large Animals

**DOI:** 10.34133/bmr.0262

**Published:** 2025-10-02

**Authors:** Zhen Shang, Nailong Pan, Xiaomin Wang, Xiao Xu, Yanhan Dong, Dan Han, Liang Zhang, Junlin Lv, Yiwei Xu, Yan Tang, Xiaotong Li, Xiaoying Kong, Wenhua Xu

**Affiliations:** ^1^ Institute of Regenerative Medicine and Laboratory Technology Innovation, Qingdao University, Qingdao, Shandong 266071, PR China.; ^2^ Qingdao Medical College, Qingdao University, Qingdao, Shandong 266071, PR China.; ^3^ School of Basic Medicine, Qingdao University, Qingdao 266071, PR China.

## Abstract

Alkali burn of corneas can induce corneal stromal fibrosis and limbal stem cell deficiency, which destroys corneal epithelial homeostasis, leading to scarring and impaired vision. Although stem cell therapy has shown potential therapeutic contributions to corneal injuries, it still faces the challenges of difficult retention and low survival rates due to the limitations of corneal curvature and an abnormal microenvironment. In this work, a 4D-printed chitosan-based hydrogel (4D-CTH) was prepared to load limbal stem cells (LSCs) for the regulation of epithelial microenvironment homeostasis and the repair of alkali-burned corneas. 4D-CTH, which has good biocompatibility and a regular spatial structure, was proven to be a candidate for use as a tissue engineering carrier that supplies highly active LSCs to a cornea injured by alkali. Both in vitro and in vivo studies confirmed that treatment with 4D-CTH + LSCs can provide more efficient corneal repair for alkali burn injuries compared to epidermal growth factor, which is the traditional treatment method for treating burned corneas. Based on single-cell sequencing analysis, 4D-CTH can markedly increase the proportion of LSCs in corneal tissue by promoting the residence and growth of LSCs. Additionally, 4D-CTH loaded with LSCs can inhibit and reverse corneal fibrosis by interfering with fibroblast differentiation, which is closely related to the down-regulation of cytochrome c oxidase subunit VIc expression by LSCs, thereby inhibiting oxidative phosphorylation in fibroblasts. In conclusion, this work not only confirmed the feasibility of 4D-CTH + LSCs for the treatment of corneas burned by alkali but also clarified the regulation mechanism of corneal epithelial homeostasis by 4D-CTH + LSCs, providing theoretical support and an application paradigm for corneal tissue engineering therapy.

## Introduction

The maintenance of corneal physiological homeostasis plays a decisive role in visual acuity. The corneal epithelium, as the first mechanical barrier against external injury, is susceptible to various physical and chemical influence [1]. Ocular burn accounts for 11.5% to 22.1% of blinding eye injuries, even as high as 29.9% in parts of countries [2]. As a common type of eye burn caused by alkali, corneal alkali burns are characterized by the penetration of the corneal stroma layer, the destruction of proteoglycans and collagen, and the saponification reaction of fatty acids in the cell membrane, all of which lead to severe corneal dysfunction. The damaged cornea further secretes proteolytic enzymes, which cause continuous damage to the surrounding tissues and lead to local ischemia and necrosis of the cornea [3]. After alkali burn, the damaged corneal stromal cells transform into more active fibroblasts, which synthesize and secrete large amounts of collagen, fibronectin, and other numerous extracellular matrix (ECM) components, thereby inducing corneal stromal fibrosis and scarring and substantially reducing vision [4]. On the other hand, severe ocular burns can lead to corneal limbal stem cell deficiency (LSCD), which further disrupts the corneal epithelium’s homeostasis [5]. Moreover, in samples of LSCD patients, corneal limbal stem cells (LSCs) exhibit a reduction in quantity, impaired proliferation function, and unsuccessful corneal epithelial repair due to their susceptibility to disturbances in the stromal microenvironment [6]. Therefore, exogenous LSC supplementation and regulation of corneal epithelial homeostasis have become the main goals of clinical treatment for LSCD.

The current strategies for exogenous LSC transplantation mainly include corneal transplantation and amniotic membrane transplantation [7]. Unfortunately, clinical data prove a poor prognosis of traditional corneal transplantation due to the patient’s autoimmune rejection reaction. Furthermore, the severe shortage of donor corneas also restricts the promotion of traditional corneal injury repair strategies. According to clinical statistics, over 98.5% of patients require donated corneas, yet less than 2% of them are able to successfully undergo corneal transplantation [8]. On the other hand, as a common method for corneal repair, amniotic membrane transplantation has several insurmountable drawbacks, including low transparency, unsatisfactory tensile strength, and considerable batch-to-batch variations. Moreover, over time, the transplanted amniotic membrane is subject to erosion and degradation, which is the primary cause of the failure of amniotic membrane transplantation [9]. Therefore, the transplantation strategy of exogenous LSCs currently faces major challenges including high donor dependence, unsatisfactory adaptation of the transplanted cell microenvironment, and lack of functional evaluation criteria [10]. In addition, although the existing new assistive technologies (such as biological scaffolds based on natural polymer materials, stem-cell-derived exosome therapy, and 3-dimensionally printed intelligent hydrogel systems) for LSCD treatment have made a lot of attempts in inhibiting inflammatory response, inhibiting angiogenesis, promoting epithelial regeneration, and regulating ECM synthesis and decomposition, they still face challenges due to the mismatch in host–guest performance and an unreasonable drug release curve, which induce serious eye complications, such as glaucoma, cataract, and corneal perforation [11]. Therefore, innovative clinical strategies are urgently needed to address the technical shortcomings of exogenous LSC transplantation strategies and LSCD neoassistive technologies in LSC donor support and corneal epithelial homeostasis regulation.

In the field of corneal tissue engineering, the choice of biomaterials plays a critical role in the control of biocompatibility, mechanical stability, and biodegradability. Hydrophilic materials such as chitosan (CTS), gelatin, hyaluronic acid, and polyethylene glycol-based hydrogels have demonstrated excellent support for cell adhesion, proliferation, and wound healing [12]. Among them, CTS and its derivatives are widely studied for their natural antibacterial activity and their ability to promote re-epithelialization and collagen remodeling during the wound healing process. Because the characteristic amino group (–NH_2_) is protonated in the physiological environment to form the positively charged –NH_3_^+^, CTS can disrupt the negative charge structure of bacterial cell membranes (such as the phospholipid bilayer) through electrostatic interaction, causing the leakage of cell contents and effectively inhibiting common pathogenic bacteria such as *Staphylococcus aureus* and *Escherichia coli* [13]. Additionally, CTS has been proven to alleviate inflammation by promoting the release of transforming growth factor-β by macrophages and down-regulating the expression of pro-inflammatory factors such as tumor necrosis factor-α, and it can also promote fibroblast proliferation and granulation tissue formation by up-regulating collagen synthesis genes. Furthermore, due to the satisfactory light transmittance, oxygen permeability, and mechanical strength and flexibility that are suitable for adapting to the curvature of the eye, CTS has become one of the ideal choices for artificial corneal materials [14]. Therefore, CTS-based hydrogels are expected to be integrated into 4-dimensional (4D) printing platforms to fabricate corneal scaffolds that can simulate the tissue remodeling process [15].

Four-dimensional bioprinting tissue engineering technology, which relies on specially intelligent materials (such as shape-memory polymers and stimulus-responsive hydrogels) and preset structural design, endows tissue engineering scaffolds with the function of controllable morphological/functional transformation under specific stimuli (temperature, pH, humidity, etc.) [16]. Four-dimensional bioprinting tissue engineering technology can achieve accurate regulation of the pore size and stiffness of hydrogel carriers and simulation of dynamic changes in the limbal microenvironment [17]. The carriers of 4D-bioprinting tissue engineering can not only provide a porous structure for stem cells, which is conducive to the adhesion, growth, migration, and proliferation of stem cells, but also simulate the mechanical properties of the natural limbus to promote the induction of direct differentiation of stem cells [18]. Our previous research studies have confirmed that a CTS-based 4D-bioprinting carrier can provide a satisfactory loading rate of autologous limbal LSCs and could provide more satisfactory stemness maintenance of stem cells than traditional hydrogels [19]. Therefore, the 4D-bioprinted tissue engineering strategy is expected to support both the transplantation of exogenous LSCs and the maintenance of corneal epithelial homeostasis, aiding the optimization of clinical treatments for LSCD.

As 2 natural polysaccharides with satisfactory biocompatibility, CTS and carboxymethyl chitosan (CMCTS), when mixed with sodium glycerophosphate in a certain ratio, induce changes in hydrogen bond forces between the polymer chains at different temperatures that endow the hydrogel with temperature-sensitive properties. Therefore, it is possible to achieve bioprinting at low temperatures and gelation at higher temperatures [19]. In this study, a 4D-printed chitosan-based hydrogel (4D-CTH) was prepared for the loading of LSCs, which were then used for the treatment of corneal alkali burn in Bama piglets. Compared with rats and New Zealand rabbits, Bama piglets could provide corneas that are more similar to human corneas with multiple structural features and biomechanical properties, making them more representative and reliable in simulating human corneal diseases and related treatments. In vivo results showed that after the 7th day of 4D-CTH + LSCs treatment, corneal turbidity was markedly lower than that in the traditional treatment group, and the epithelial healing rate of cornea was substantially faster than that in the traditional epidermal growth factor (EGF) treatment group. In order to explore the repair mechanism of burned corneas by 4D-CTH + LSCs, corneal tissues after 7 d of treatment were analyzed by single-cell sequencing. Cluster analysis confirmed that 4D-CTH markedly up-regulated the proportion of LSCs in corneal tissue while down-regulating the proportion of fibrosis-related cells. Monocle2 quasi-temporal analysis and RNA rate analysis (scVelo) revealed the inhibition of fibroblast activation in the 4D-CTH + LSCs group, suggesting that 4D-CTH + LSCs may effectively inhibit or reverse corneal fibrosis by regulating cell–cell interactions and signaling. Cell communication analysis of CellPhoneDB indicated that the microenvironmental conditions provided by 4D-CTH could enhance the regulation of LSCs on fibroblasts. Finally, Kyoto Encyclopedia of Genes and Genomes (KEGG) and gene set enrichment analysis (GSEA) clarified that LSCs in 4D-CTH reverse the fibrosis of damaged cornea by down-regulating the oxidative phosphorylation pathway regulated by the cytochrome c oxidase subunit VIc (COX6C) gene. This study not only verified the safety and effectiveness of 4D-CTH + LSCs in the treatment of LSCD in large animals but also analyzed the dual-action mechanism of the 4D-CTH + LSCs strategy in promoting epithelial regeneration and inhibiting fibrosis, providing a generalized application paradigm for new clinical treatment strategies for LSCD.

## Materials and Methods

### Animals

Bama miniature pigs (25.0 ± 5.0 kg) aged 8 to 12 weeks were purchased from the Experimental Animal Research Institute of Sichuan Provincial People’s Hospital (Chengdu) and raised in the Experimental Animal Center of Sichuan Provincial People’s Hospital. All procedures involving animals were approved by the Medical Ethics Committee of the Experimental Animal Research Institute of Sichuan Academy of Medical Sciences & Sichuan Provincial People’s Hospital (approval number: Lunshen 2024 No. 001) and conducted in strict accordance with the Chinese Animal Administration regulations and the Association for Research in Vision and Ophthalmology Statement for the Use of Animals in Ophthalmic and Vision Research.

### Extraction and separation of primary autologous LSCs

Miniature pigs were anesthetized with sufentanil (2.5 μg/kg) and maintained on 5% isoflurane. After removing periocular hair (including eyelashes) and disinfecting the skin with 10% iodine tincture diluted 1:10 in 0.9% sodium chloride, the conjunctival sac was rinsed 3 times with phosphate-buffered saline (PBS). Under sterile conditions with a surgical drape and a lid opener, the bulbar conjunctiva was incised in an “L” shape at the fornix, and the tissue was carefully separated from the limbus. Using an ophthalmic surgical microscope, a wedge-shaped limbal tissue (approximately 2 mm × 6 mm × 0.2 mm) was excised with a tunnel knife. The specimen was immediately placed in D-Hank’s solution containing 1% triple antibiotic and soaked for 30 min, followed by enzymatic digestion (dispase II and 0.25% trypsin) to obtain single-cell suspensions [20].

### Preparation of the 4D-CTH carrier

CTS (degree of deacetylation ≥95%; viscosity 100 to 200 mPa·s) was purchased from Shanghai Maclean Biochemical Co., Ltd. (Shanghai, China). CMCTS (degree of deacetylation 96.44%, 134 kDa) was purchased and prepared according to the previous description. β-Glycerophosphate disodium was purchased from Shanghai Soraby Biotechnology Co., Ltd. Under aseptic conditions, 0.2 g of CTS was dissolved in 4.5 ml of acetic acid solution (0.1 mM) and stirred for 10 h. Then, 0.2 g of CMCTS was dissolved in 4.5 ml of double-distilled water (4.4%); β-glycerophosphate disodium was also dissolved in 2 ml of double-distilled water (6%). β-Glycerophosphate disodium solution was added to CMCTS solution (drop-by-drop addition; performed at 4 °C). After that, CTS solution was mixed with this solution, and the combined material was named CTH. CTH was printed into a homogeneous carrier by using a 4D bioprinter (Bio-Architect SR, Regenovo, China) stacking layer by layer at a low temperature (0 to 4 °C). Then, the materials were stored by undergoing vacuum freeze-drying.

### Fabrication of a 4D-printed hydrogel scaffold

The hydrogel scaffold was fabricated using a multilayer extrusion-based bioprinter (Regenovo 3D Bio-Architect WS). The printing resolution and process parameters were set according to slicing specifications. Specifically, segment 1 was printed with a layer height of 0.11 mm (2 layers, total 0.22 mm), and segment 2 with 0.116 mm (2 layers, total 0.232 mm), resulting in a total structure height of 0.452 mm. The final model dimensions were 20 mm × 20 mm × 3 mm (*X* × *Y* × *Z*).

For extrusion, segment 1 was printed at 0.03 MPa and 12 mm/s, while segment 2 used the same air pressure but a speed of 14 mm/s, both using a linear infill pattern. Multilayer infill printing was performed with a randomized starting point to avoid seam overlap.

These settings ensured structural precision and consistency, which are crucial for the subsequent shape-morphing behavior of the 4D scaffold. The actuation trigger for the 4D shape change is temperature-responsive swelling and deswelling in an aqueous environment at 37 °C. The shape-memory behavior is governed by the thermosensitive polymer network of the CTH hydrogel, which undergoes reversible deformation in response to temperature change (37 °C to room temperature) and moisture uptake. This allows the printed scaffold to self-adjust its curvature to match the corneal surface after implantation.

### Physical characterization of 4D-CTH

The attenuated total reflectance Fourier transform infrared (FTIR) spectra of the 4D-CTH hydrogel were recorded using a Nicolet iS50 FTIR spectrometer (Thermo Scientific, Waltham, MA, USA) over a wave number range of 400 to 4,000 cm^−1^. The samples were first frozen and lyophilized, then ground into fine powder, and mixed with KBr for pellet preparation. Data acquisition and spectral analysis were performed using the OriginPro 9.1 software. To observe the microstructure of the hydrogel, the 4D-CTH and CTH scaffolds were subjected to vacuum freeze-drying and then sputter-coated with gold. The surface and internal pore morphology were examined using a scanning electron microscope (Tescan, Brno, Czech Republic). The images were used to assess pore size distribution and porosity. Porosity measurements were quantified from the scanning electron microscopy images using the ImageJ software.

### Antibacterial activity assessment of 4D-CTH

The antibacterial efficacy of the CTS thermosensitive hydrogel and 4D-CTH against representative Gram-positive (*S. aureus*) and Gram-negative (*E. coli*) bacteria was evaluated. Briefly, 600 mg of sterilized hydrogel and 600 mg of 4D-CTH were placed in 24-well plates and evenly distributed on the well bottoms. Subsequently, 10 μl of bacterial suspension (10^6^ colony-forming units [CFU]/ml in PBS) was carefully applied to the surface of each hydrogel sample. The samples were incubated at 37 °C for 4 h under >90% relative humidity. Following incubation, 1 ml of sterile PBS was added to each well to resuspend the surviving bacteria. A bacterial suspension in PBS without hydrogel served as the positive control. Then, 100 μl of each bacterial suspension was plated onto Columbia blood agar and incubated at 37 °C for 18 to 24 h. CFUs were enumerated thereafter. All experiments were performed in triplicate. The antibacterial rate was calculated as

$$\text{Inhibition rate}\;\left(\%\right)=\frac{C_{0}-C_{s}}{C_{0}\;}\times 100\%$$ (1)

where *C*_0_ and *C_s_* denote the CFU counts of the control and treated groups, respectively.

### In vitro degradation of 4D-CTH

To ensure compatibility with the corneal environment, the in vitro degradation of 4D-CTH was evaluated in a simulated tear fluid containing lysozyme (1.5 mg/ml, Biosharp, China), with a pH of 7.4. The hydrogels were incubated at 37 °C, and the mass loss rate was determined at 5 predefined time points, days 0, 1, 3, 5, and 7, using the dry weight method. The pH of the simulated tear fluid was maintained at 7.4 throughout the experiment.

### In vitro cytocompatibility of 4D-CTH

To evaluate the in vitro cytocompatibility of the 4D-CTH scaffold, both live/dead cell staining and 3-(4,5-dimethylthiazol-2-yl)-2,5-diphenyltetrazolium bromide (MTT) assays were performed using porcine corneal epithelial cells (CECs) preserved in our laboratory. The cells were isolated from the corneal tissue of healthy adult Bama miniature pigs (with ethical approval) using the tissue explant adhesion method and cultured under standard conditions at 37 °C in a humidified incubator with 5% CO_2_, using Dulbecco’s modified Eagle medium (DMEM)/F12 medium (Gibco, USA) supplemented with 10% fetal bovine serum (Gibco, USA) and 1% penicillin–streptomycin (Solarbio, Beijing, China).

According to the ISO 10993-5 guidelines, 4D-CTH scaffolds were immersed in DMEM/F12 medium for 24 h, and the resulting extraction solution was collected as the treatment medium. Porcine CECs were seeded into 12-well plates at a density of 1 × 10^5^ cells/well and cultured for 24 h under standard conditions. The culture medium was then replaced with the 4D-CTH extract, and cells were incubated for an additional 24 h. After treatment, cells were gently washed with PBS and stained using a calcein-AM/propidium iodide (PI) double staining kit (Dalian Meilun Biotechnology Co., Ltd., China) following the manufacturer’s instructions. Briefly, 4 μl of calcein-AM and 4 μl of PI were mixed with 4 ml of PBS to prepare the working solution. The cells were incubated in the staining solution for 15 min at room temperature in the dark. Fluorescence images were acquired using an inverted fluorescence microscope (Olympus, Japan).

### In vitro cytotoxicity assessment

The material was soaked in DMEM/F12 medium for 24 and 48 h, respectively, and the extraction solutions were collected as treatment samples. CECs were seeded into 96-well plates and cultured until the logarithmic growth phase. The culture medium was then replaced with the extract solutions from different time points, and cells were further incubated for 24 h under the same conditions. Cell viability was assessed using the MTT assay. MTT solution (final concentration 0.5%) was added to each well and incubated at 37 °C for 4 h. After discarding the supernatant, dimethyl sulfoxide was added to dissolve formazan crystals, followed by thorough shaking. The optical density was measured at 490 nm, and cell viability was calculated accordingly.

### Primary culture of LSCs

The collected limbal tissue was placed in 1.5 U/ml dispase II (Sigma-Aldrich, USA) and incubated at 4 °C for 14 to 16 h. Subsequently, the epithelial cell layer and the stromal layer were gently separated by mechanical separation, followed by digestion with 0.25% trypsin (Thermo Fisher Scientific, USA) at 37 °C for 5 min. The obtained single-cell suspension was inoculated in mesenchymal stem cell basal medium (Dakewe, Shenzhen, China) containing 5% SUPERGROW Cell Culture Supplement (Dakewe, Shenzhen, China) and 1% penicillin–streptomycin (Solarbio, Beijing, China) for primary culture, and fresh medium was replaced every 48 h. When the cell confluence reached 80% to 90%, trypsin was used for digestion and passage. In this experiment, LSCs of the first to third generations were selected for subsequent analysis.

### Identification of the stemness of LSCs

The stemness of the isolated LSCs was confirmed by immunofluorescence staining for B lymphoma Mo-MLV insertion region 1 homolog (BMI1), Delta Np63 alpha (ΔNp63α), cytokeratin 14 (CK14), and ATP-binding cassette sub-family G member 2 (ABCG2). Only cells from passages 1 to 3 were used for subsequent experiments to ensure preservation of stem cell characteristics. Sterile cell slides (Thermo Fisher Scientific, USA) were placed in 6-well plates (Corning, USA) and coated with LN521 solution (BioLamina, Sweden). Subsequently, P1 and P2 cells were seeded onto the treated slides at a density of 60% to 70%. After sealing, the plates were fixed with 4% paraformaldehyde fixative (Sigma-Aldrich, USA) at room temperature for 20 min, followed by permeabilization with 0.2% Triton X-100 (Sigma-Aldrich, USA) for 5 min. After blocking for 30 min, the following specific primary antibodies were added and incubated overnight at 4 °C: BMI1 antibody (Cell Signaling Technology, USA), ΔNp63α antibody (Cell Signaling Technology, USA), CK14 antibody (Cell Signaling Technology, USA), and ABCG2 antibody (Proteintech, China). Then, Alexa Fluor 555-labeled anti-mouse immunoglobulin G secondary antibody and Alexa Fluor 488-labeled anti-rabbit immunoglobulin G secondary antibody (Cell Signaling Technology, USA) were added and incubated at room temperature for 1 h in the dark. 4′,6-Diamidino-2-phenylindole staining solution (Solarbio, Beijing, China) was used for 10 min, and 20 μl of ProLong Gold antifluorescence quencher (Cell Signaling Technology, USA) was added, and the images were observed and collected using a fluorescence microscope (Nikon, Japan).

### Construction of a corneal alkali burn model

Before the experiment, miniature pigs received preoperative general anesthesia (intravenous sufentanil, 2.5 μg/kg, Humanwell, Yichang, China) in a sterile operating room and continued to receive air anesthesia (5% isoflurane) during the operation. Subsequently, the pigs’ eyelids and surrounding areas were disinfected using cotton swabs dipped in 75% alcohol, and the ocular surface was topically anesthetized using proparacaine hydrochloride eye drops (15 ml:75 mg, Alkain, Beijing, China). Next, an 8-mm Whatman no. 1 filter paper soaked in 1 M NaOH was placed on the right cornea of each pig for 60 s, after which the cornea was thoroughly rinsed with 0.9% saline to remove excess NaOH. In addition, pigs were given buprenorphine (0.05 mg/kg, Orion Parma, Finland) to relieve discomfort at the end of the surgery and when signs of pain and discomfort were observed during the recovery process. In the experiment, the miniature pigs were randomly divided into 5 groups, with the right eyes for modeling and the left eyes as self-control. Model control group: only PBS was used to rinse the alkali-burned ocular surface; positive control group: EGF gel was applied to the corneal burn site; 4D-CTH group: the carrier melted at room temperature for 15 to 20 s was completely attached to the corneal burn site; LSCs group: 200 μl of LSC single-cell suspension with a concentration of 3 × 10^6^ cells/ml was dripped on the burn site; 4D-CTH + LSCs group: the melted 4D-CTH carrier was first attached to the corneal burn site, and then an equal amount of LSC suspension was dripped onto the burn site. After the treatment, the pig eyes were photographed and recorded, and then the eyelids were sutured. After surgery, 0.3% ofloxacin eye drops were given daily to prevent infection.

### Observation of the external ocular images of the alkali burn model

On the 3rd day after surgery, the eyelid sutures were removed. The ocular surface of the burns in each group was observed on days 0, 3, 7, 14, and 21, and the corneal transparency and epithelial defect healing were recorded and photographed. The corneal transparency score was based on the following criteria: 0 level, completely transparent cornea; 1st level, mild corneal opacity; 2nd level, mild stromal opacity (visible pupil edge and iris blood vessels); 3rd level, moderate stromal opacity (only visible pupil edge); 4th level, severe stromal opacity (visible anterior chamber); and 5th level, complete corneal opacity (anterior chamber not visible). In addition, all experimental eyes were stained with sodium fluorescein ophthalmic test strips and photographed under slit lamp blue light. Finally, all corneal epithelial photos were analyzed uniformly using the ImageJ software.

### Histology and immunohistochemical staining

All animals were euthanized 21 d after surgery in accordance with animal welfare requirements. The removed eyeballs were embedded in paraffin and cut into sections with a thickness of 5 μm. First, hematoxylin and eosin (HE) or Masson trichrome staining (Sigma, USA) was used for staining according to the manufacturer’s instructions. The samples were then given immunohistochemical staining. Specifically, the sample was first sealed at room temperature with 5% goat serum for 1 h. The samples were then incubated overnight at 4 °C for alpha smooth muscle actin (α-SMA; Abcam, Cambridge, UK) or Ki67 primary antibody (Abcam, Cambridge, UK) and at room temperature for 1 h for the corresponding secondary antibody (1:2,000, Abcam). After staining, the sections were observed and photographed using a fluorescence microscope, and the following indicators were quantitatively analyzed using the ImageJ software: corneal epithelial thickness, matrix collagen volume percentage, Ki67-positive rate of CECs, and α-SMA-positive area.

### Corneal irritation/anti-inflammatory activity evaluation

To evaluate the inflammatory response and anti-inflammatory effects of the material during corneal injury repair, corneal tissue specimens were collected from the 4D-CTH + LSCs group on day 7 (D7) after a corneal alkali burn injury model. Paraffin sections were prepared and immunohistochemically stained. The expression of the inflammatory cell marker CD11b and the anti-inflammatory factor interleukin-10 (IL-10) was detected, and the staining results were observed and analyzed under a microscope.

### In vivo degradation and residue analysis

To assess in vivo degradation and material retention, paraffin-embedded corneal tissues were collected from the alkali-burned cornea model on D7 and day 28 (D28) posttreatment. Tissue sections were prepared and stained with Alcian Blue to detect and analyze the distribution and residual presence of CTS within the corneal tissue.

### 10× Genomics single-cell RNA sequencing

Each sample was processed for single-cell isolation to prepare a single-cell suspension, and then the 10× Genomics system was used for library construction. First, GEM (gel bead emulsion) was generated by cell lysis to construct a barcoded complementary DNA library. All steps were strictly performed according to the manufacturer’s instructions for Single Cell 3’ mRNA Kit (V2; 10× Genomics). Finally, all constructed libraries were subjected to rigorous quality assessment before subsequent sequencing analysis could be performed.

### Cell cycle differential analysis

Cell-cycle-specific expression profiles were used to determine the cell cycle phase of each cell in Seurat. G2/M and S phase markers were used to score cells, where cells lacking G2/M and S phase markers were classified as G1 phase (CellCycleScoring function). The prop.table function was used to quantify the cells within each stage.

### Pseudotime analysis

The Monocle2 software was used for quasi-time series analysis of single-cell samples. Single-cell RNA sequencing (scRNA-seq) data were modeled as a manifold in a high-dimensional space, enabling the identification of each cell’s transcriptional state and transformation path. For the Monocle2 software, dimensionality reduction algorithms such as t-distributed stochastic neighbor embedding or principal component analysis were used to reduce high-dimensional data to 2 or 3 dimensions for visualization and comparison. Simultaneous hidden Markov models and dynamic time warping were employed to characterize the expression profiles of individual cells on a timeline.

### RNA velocity analysis

scVelo was used to analyze the dynamic changes of unclipped and clipped messenger RNA (mRNA) in the single-cell transcriptome to infer the direction of cell state transition. The abundance ratio of uncut mRNA (representing nascent transcripts) to cut mRNA (representing mature transcripts) was used to calculate the RNA velocity vector. Dynamic models were adopted and dynamic driver genes were systematically identified to analyze the full information on the splicing dynamics of each gene to find the key drivers that control cell fate transformation.

### Cell–cell communication analysis

The CellPhoneDB software (version 1.1.0, https://github.com/Teichlab/cellphonedb) was used to systematically predict and analyze cell–cell interactions, and then the ligand–receptor interaction was evaluated using the default parameter settings. The key receptor–ligand pairs obtained after screening were finally visualized using the Circlize R software package to clearly display the network relationship of cell–cell interactions.

### Statistical analysis

Statistical analyses were performed using GraphPad Prism. Depending on the data type, appropriate statistical tests such as 2-tailed *t* tests or one-way analysis of variance (ANOVA) were used. For gene set score analysis, 2-sided Wilcoxon rank-sum tests were applied. *P* values less than 0.05 were considered statistically significant. The symbols *, **, ***, and **** indicate *P* < 0.05, 0.01, 0.001, and 0.0001, respectively.

Post hoc power analyses were conducted based on key outcome indicators to assess the adequacy of the sample size used in the in vivo studies. Although 4 animals were assigned to each group, 3 were included in the final analysis to ensure data consistency and minimize intersample variability. Power analysis was performed with *n* = 3 using the G*Power software (version 3.1). For CD11b expression levels, a 2-tailed *t* test comparing the untreated and combined treatment groups yielded a power of 0.86 (effect size = 2.61 and *α* = 0.05). For corneal epithelial healing quantification on D7, one-way ANOVA across 5 groups showed a power of 0.83 (effect size *f* = 1.48 and *α* = 0.05). These results indicate that the sample size was sufficient to detect statistically significant differences in the primary outcomes.

## Results

### Preparation and physical characterization of 4D-CTH

**Fig. 1.**
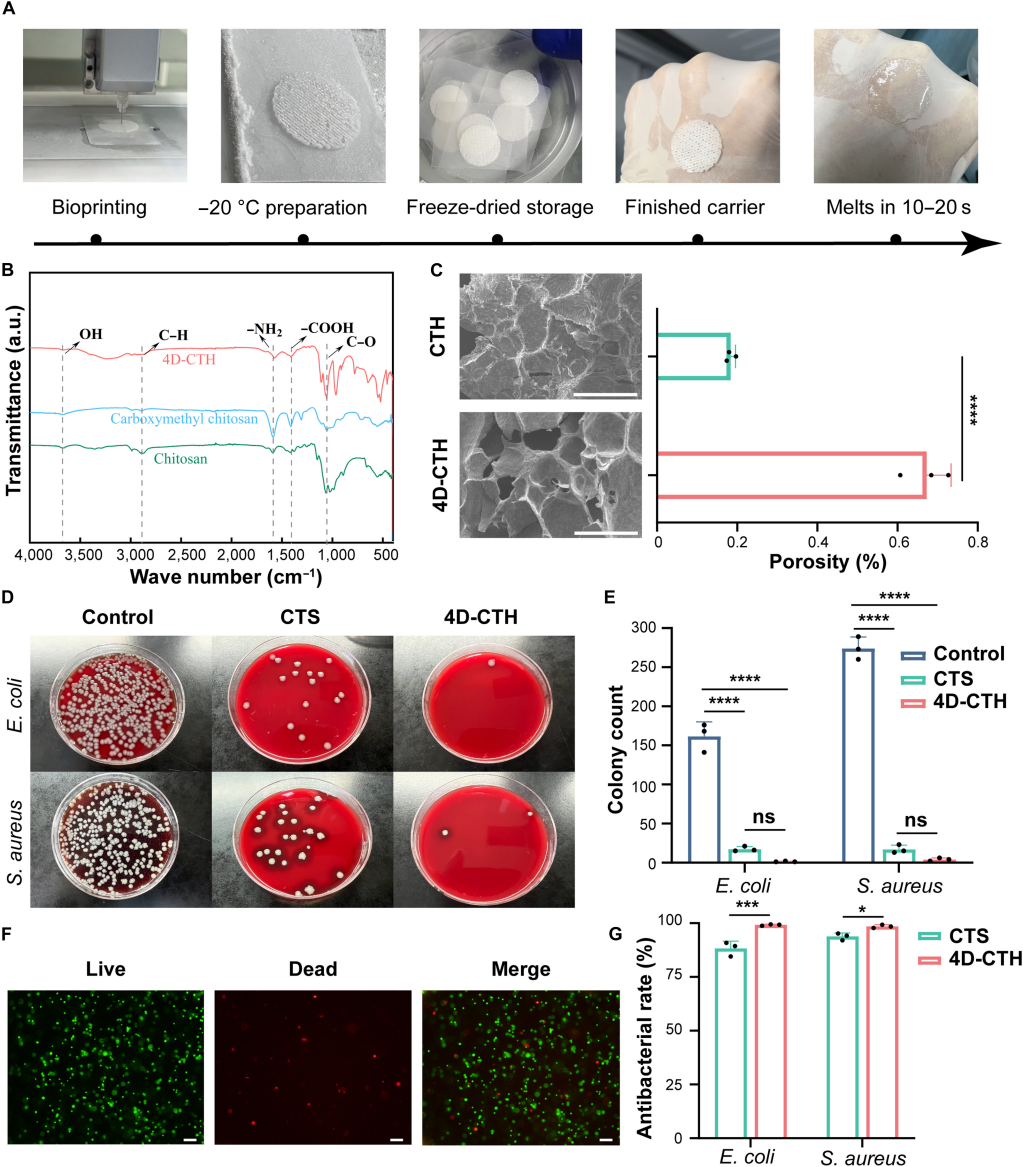
Preparation and performance characterization of 4D-printed chitosan-based hydrogel (4D-CTH). (A) Preparation of 4D-CTH and observation of skin adhesion. (B) Infrared spectra of 4D-CTH, chitosan, and carboxymethyl chitosan. (C) Scanning electron microscopy (SEM) observation of 4D-CTH and CTH (left) and gel porosity (right); scale bar: 100 μm. (D and E) Antibacterial observation and colony count of CTH and 4D-CTH against Gram-positive bacteria (*Staphylococcus aureus*) and Gram-negative bacteria (*Escherichia coli*). (F) Cell viability detection after 4D-CTH-encapsulated adipose-derived mesenchymal stem cells. Scale bar: 200 μm. (G) Antibacterial efficiency of CTH and 4D-CTH against Gram-positive bacteria (*S. aureus*) and Gram-negative bacteria (*E. coli*). *P < 0.05; ***P < 0.001; ****P < 0.0001; ns, not significant. CTS, chitosan.

The prepared 4D-CTH has been confirmed to have satisfactory temperature sensitivity and viscoelasticity in previous studies [14]. After the solid 4D-CTH was adhered to the skin surface, it was found that 4D-CTH transformed from the solid state to the gel state and achieved a more firm and tight adhesion after 10 to 15 s (Fig. 1A). The FTIR spectroscopy results of 4D-CTH showed that the material exhibited a broad absorption band corresponding to hydroxyl (–OH) groups near 3,500 cm^−1^, a stretching vibration peak of C–H near 2,900 cm^−1^, and a bending vibration peak of amino (–NH_2_) groups near 1,600 cm^−1^, confirming the presence of CTS in CTH. Additionally, the peaks near 1,500 and 1,000 cm^−1^ were attributed to the stretching vibrations of carboxyl (–COOH) groups and C–O, indicating the presence of CMCTS (Fig. 1B) [21]. Scanning electron microscopy images revealed the pore characteristics of 2 CTS hydrogels, 4D-CTH and CTH. Compared with CTH, 4D-CTH showed a more uniform pore distribution and a higher porosity (*P* < 0.05) (Fig. 1C), which is of great significance for cell loading and migration. In addition, CTH and 4D-CTH demonstrated satisfactory antibacterial performance against Gram-positive bacteria (*S. aureus*) and Gram-negative bacteria (*E. coli*). Figure 1D shows that the antibacterial rates against *S. aureus* and *E. coli* of 4D-CTH were 98.52% and 99.18%, respectively (Fig. 1E and G). As shown in Fig. 1F, live/dead cell staining confirmed that the encapsulated CECs exhibited high viability and were uniformly distributed throughout the 4D-CTH hydrogel scaffold, indicating good cytocompatibility and homogeneous encapsulation. In vitro degradation analysis demonstrated that 4D-CTH underwent gradual and approximately linear degradation under physiological conditions, with substantial degradation completed by D7 (Fig. S1A). To assess the cytocompatibility of the 4D-CTH scaffold, MTT assays were performed using primary CECs. The results showed that cells cultured with 4D-CTH extracts exhibited no significant reduction in cell viability compared to the control group over the tested period (*P* > 0.05), indicating excellent biocompatibility and no cytotoxic effects (Fig. S1B).

### In vitro culture of LSCs and biocompatibility evaluation of 4D-CTH

**Fig. 2.**
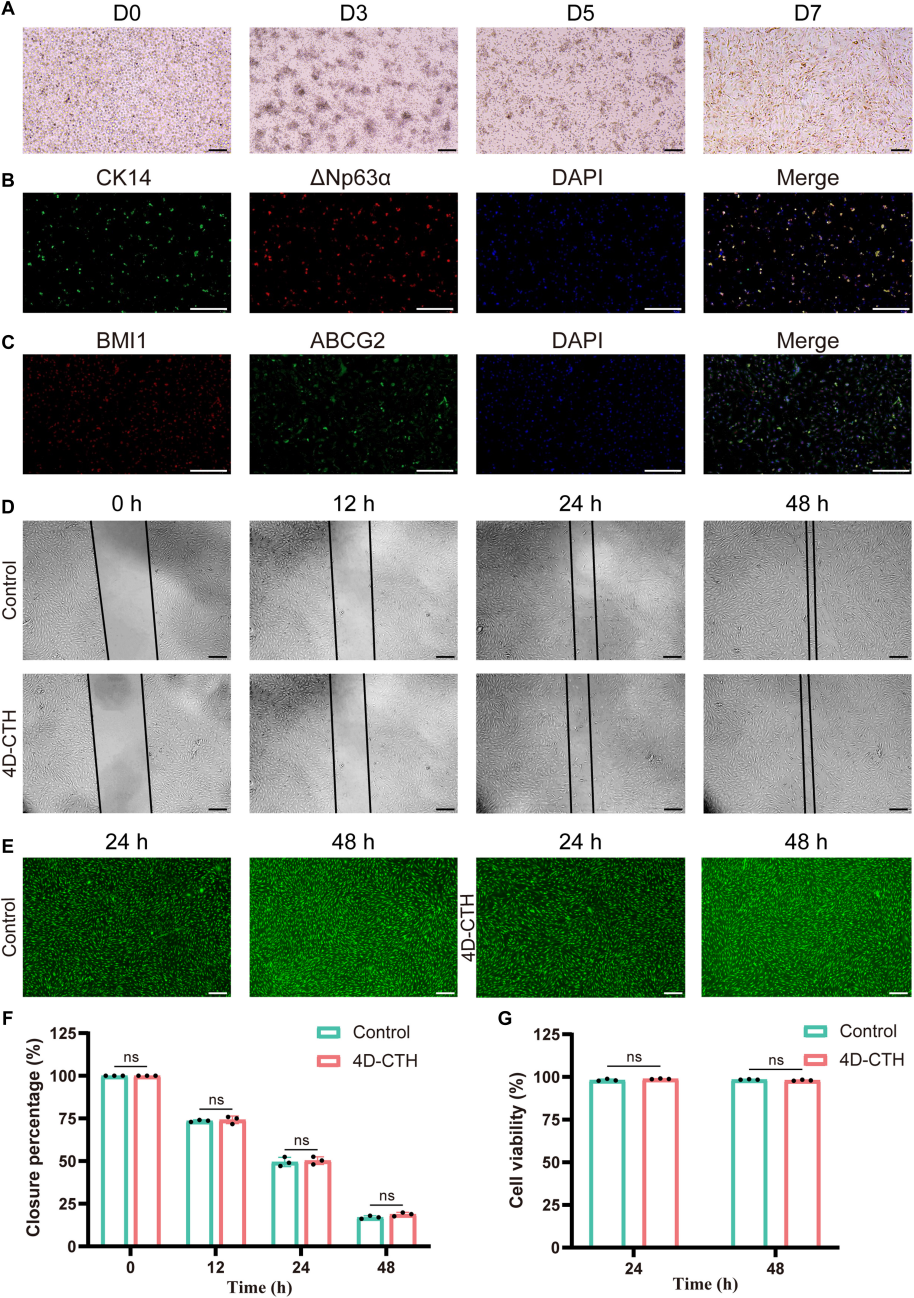
Identification of the stemness of porcine limbal stem cells (PLSCs) and evaluation of the biocompatibility of 4D-CTH. (A) Representative images of primary culture of PLSCs at different time points. (B) Dual fluorescence staining of cytokeratin 14 (CK14) and Delta Np63 alpha (ΔNp63α) to identify primary PLSCs. (C) Dual fluorescence staining of B lymphoma Mo-MLV insertion region 1 homolog (BMI1) and ATP-binding cassette sub-family G member 2 (ABCG2) to identify primary PLSCs. (D) Migration ability of 4D-CTH-encapsulated limbal stem cells (LSCs) in the flat scratch assay. (E) Representative images of live and dead cell staining of 4D-CTH- and CTH-encapsulated LSCs. (F) Quantitative analysis of healing rate in the flat scratch assay. (G) Quantitative analysis of cell viability after 4D-CTH- and CTH-encapsulated LSCs. Scale bar, 50 μm; white scale bar, 200 μm. ns, not significant. D0, D3, D5, and D7, days 0, 3, 5, and 7; DAPI, 4′,6-diamidino-2-phenylindole.

On the 1st day of the in vitro culture of porcine limbal stem cells (PLSCs) obtained by dispase II digestion, the PLSCs were observed to be round or oval and presented a semisuspended state under an inverted microscope. On the 3rd day of in vitro culture, PLSCs began to aggregate and form multiple cell clusters with surrounding cells, thereby forming stem cell islands, which were a direct evidence of the self-renewal and differentiation of PLSCs [22]. From the 5th to the 7th day of in vitro culture, the cells in the stem cell island gradually extended outward and gradually fused into a complete “pebble”-shaped cell layer (Fig. 2A). Immunofluorescence staining of specific marker molecules of LSCs, including CK14, ΔNp63α, BMI1, and ABCG2, was used for the identification of PLSCs [23,24]. The co-staining results of CK14 and ΔNp63α, as well as BMI1 and ABCG2, indicated the positive expression of the above 4 markers, confirming the successful identification and the maintenance of the stem cell characteristics of PLSCs during the in vitro culture (Fig. 2B and C). In addition, wound healing of PLSCs confirmed that PLSCs cultured on 4D-CTH had no difference in cell proliferation and migration ability compared with the control group (Fig. 2D and F). The calcein-AM/PI staining results confirmed that 4D-CTH encapsulation had no effect on the cell viability of PLSCs (Fig. 2E and G), indicating the good cytocompatibility and satisfactory biosafety of 4D-CTH.

### Combined treatment of 4D-CTH and LSCs promotes corneal regeneration and repair after alkali burn

**Fig. 3.**
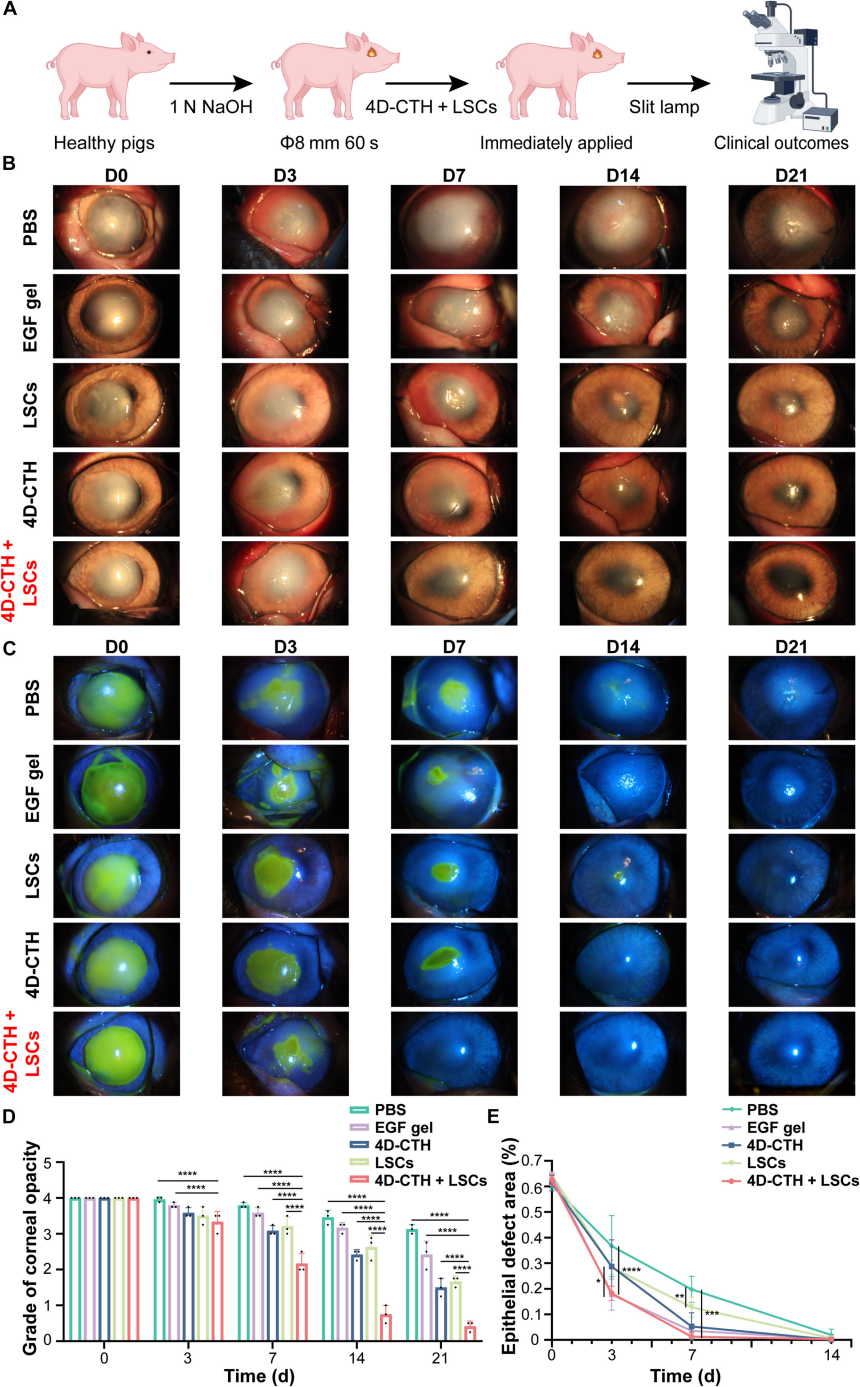
Evaluation of the effect of 4D-CTH combined with LSCs in the treatment of corneal alkali burns. (A) Schematic diagram of the construction of the corneal alkali burn model of Bama miniature pigs. (B) Slit lamp microscopy was used to observe the recovery of corneal transparency in different treatment groups. (C) Fluorescein sodium staining showing the repair of corneal epithelial defects (green fluorescence marked the defect area). (D) Corneal turbidity score statistics. (E) Statistics of corneal epithelial healing rate. *P < 0.05; **P < 0.01; ***P < 0.001; ****P < 0.0001; ns, not significant. D14 and D21, days 14 and 21; PBS, phosphate-buffered saline; EGF, epidermal growth factor.

According to the schematic diagram in Fig. 3A and Fig. S2A, the corneal alkali burn model of the right eye in Bama miniature pigs was successfully constructed, and the model pigs were randomly divided into the following groups: the PBS group, the EGF gel group, the LSCs group, the 4D-CTH group, and the 4D-CTH + LSCs group. After 4D-CTH was completely attached to the corneal burn area, LSC suspension (1.0 to 2.0 × 10^6^/ml) was added dropwise (Fig. S1B and C). During a 21-d cycle, the observation and image recording of corneal repair were regularly evaluated by slit lamp microscopy (on days 1, 3, 7, 14, and 21). As shown in Fig. 3B and C, on the 7th day of treatment, although the corneal epithelial repair in each treatment group was markedly faster than that in the control group, only the corneal transparency in the 4D-CTH + LSCs group was close to the normal level (Fig. 3D). The corneal epithelial repair of all experimental pigs was completed at on 14th and 21st days (Fig. 3E), but still only the corneal transparency of the 4D-CTH + LSCs group returned to the normal level. The above results suggested that the participation of EGF, LSCs, and 4D-CTH all contributed to the proliferation of CECs and the realization of corneal completivity. However, the reproduction of the regular and orderly 3-dimensional (3D) structure of the cornea, which is as important as corneal integrity for the recovery of normal corneal function, could be achieved only with the simultaneous participation of LSCs and 4D-CTH [25]. Specifically, LSCs are mainly responsible for the proliferation of corneal stem cells, while 4D-CTH contributes to providing a standardized and precise path for the migration of LSCs, thereby establishing a new corneal tissue.

**Fig. 4.**
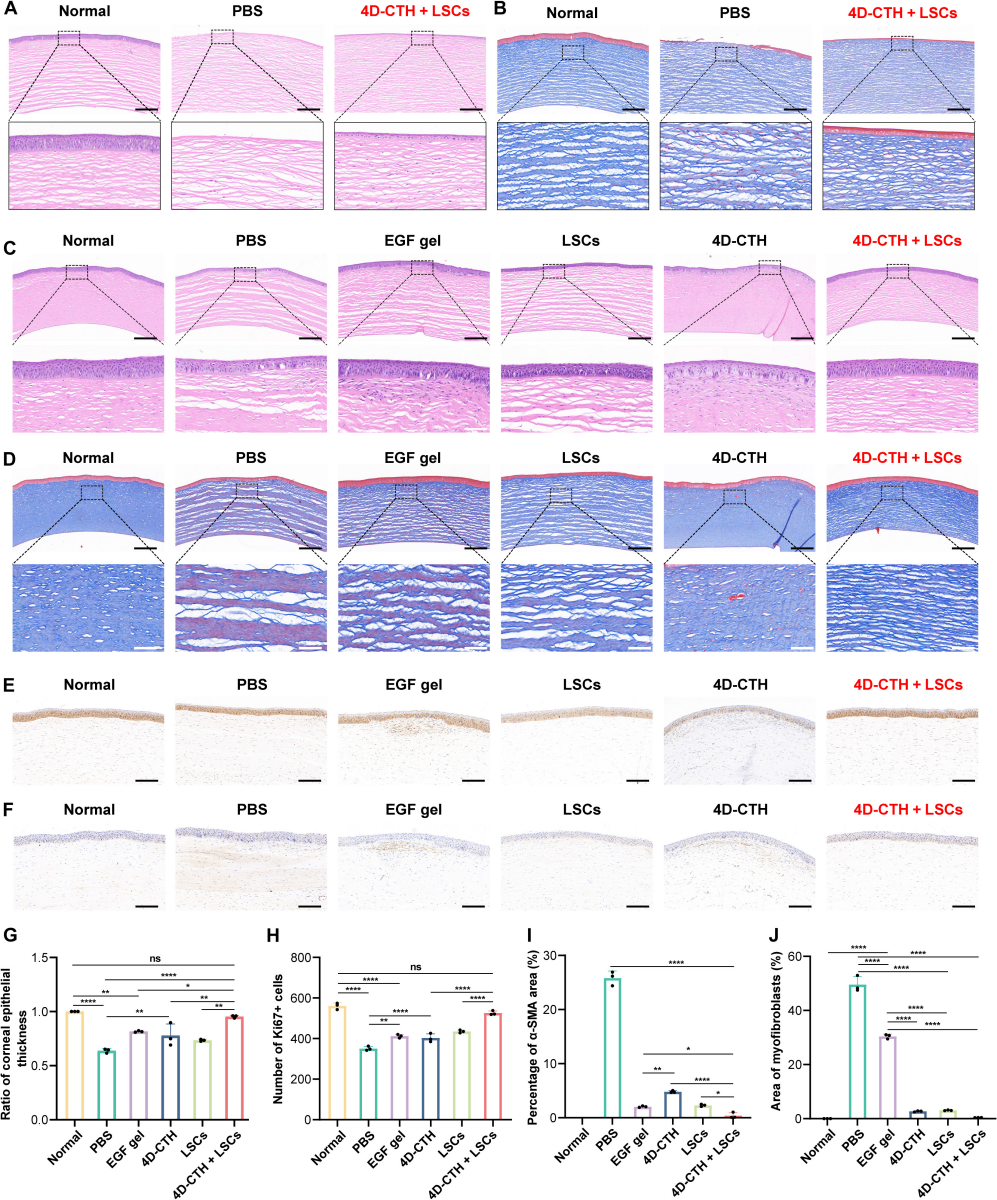
Histological evaluation of 4D-CTH combined with LSCs in the treatment of corneal alkali burns. (A) Hematoxylin and eosin (HE) staining images of corneal tissue on D7 after alkali burns. (B) Masson trichrome staining images of corneal tissue on D7 after alkali burns. (C) HE staining images of corneal alkali burns treated for 21 d. (D) Masson trichrome staining images on D21 after corneal alkali burns. (E) Ki67 immunohistochemical staining images on D21 after corneal alkali burns. (F) Representative images of alpha smooth muscle actin (α-SMA) immunohistochemical staining on D21 after corneal alkali burns. (G) Quantitative analysis of HE staining on D7 after corneal alkali burns. (H) Quantitative analysis of Ki67-positive rate on D21 after corneal alkali burns. (I) Quantitative analysis of α-SMA-positive rate on D21 after corneal alkali burns. (J) Quantitative analysis of Masson trichrome staining on D7 after corneal alkali burns. Black scale bar, 100 µm; white scale bar, 20 µm. *P < 0.05; **P < 0.01; ***P < 0.001; ****P < 0.0001; ns, not significant.

The histological staining results confirmed the above analysis and conclusions. On the 7th day, the 4D-CTH + LSCs group showed more marked advantages than the control group and other treatment groups in corneal tissue repair and fibrosis inhibition. HE staining showed that on the 7th day, the corneal tissue structure of the 4D-CTH + LSCs group was substantially improved compared with that of the control group, which was specifically manifested as the restoration of normal epithelial layer thickness, reduced stromal layer edema, and a more regular arrangement of endocortical cells (Fig. 4A). Masson tricolor staining further indicated that compared with that in the control group, the corneal tissue in the 4D-CTH + LSCs group exhibited a more regular arrangement of collagen fibers, higher collagen maturity, and milder fibrosis on the 7th day (Fig. 4B). These results indicated that 4D-CTH + LSCs treatment can markedly promote corneal tissue repair and inhibit fibrosis in the early stage of alkali burns. The histological results on the 21st day further confirmed the effect of 4D-CTH + LSCs treatment in promoting corneal repair and antifibrosis. HE staining showed that the stromal layer edema of corneal tissue in the 4D-CTH + LSCs group had completely disappeared on the 21st day, and the regularity of endocortical cells and the thickness of the corneal epithelium were significantly higher than those in the other treatment groups (*P* < 0.05, *) (Fig. 4C and G). Masson staining indicated that on the 21st day, the 4D-CTH + LSCs group presented a closely ordered arrangement of collagen fibers and significantly higher matrix collagen content and myofibroblast area in the matrix layer (*P* < 0.05, *) (Fig. 4D and H). In addition, the positive rate of Ki67 in the 4D-CTH + LSCs group was significantly higher than those in the other groups (*P* < 0.05, *), indicating that treatment with 4D-CTH + LSCs could considerably promote the proliferation of CECs and activate the epithelial regeneration and repair function, thereby accelerating corneal healing. Moreover, the number of α-SMA-positive cells in the 4D-CTH + LSCs group was significantly lower than those in the other groups (*P* < 0.05, *), which confirmed that 4D-CTH + LSCs treatment could markedly alleviate corneal fibrosis by inhibiting the activation of fibrosis-related cells (Fig. 4E to J). HE staining analysis of the hearts, livers, spleens, lungs, and kidneys of the miniature pigs showed that there were no significant histological differences between the normal group and the 4D-CTH + LSCs group (Fig. S3). Therefore, the above results confirm that, as an ideal tissue engineering carrier, 4D-CTH, which has satisfactory cell compatibility and tissue compatibility, can provide an ideal environment for the survival and maintenance of stem cells’ stemness, thereby substantially promoting the repair of the alkali-burned cornea by LSCs. In vivo degradation was further confirmed by Alcian Blue staining of corneal tissues on both D7 and D28, which showed no detectable residual CTS in the corneal epithelium at either time point, indicating complete degradation of the scaffold (Fig. S4C). Immunohistochemical staining of corneal sections on D7 posttreatment revealed reduced expression of the pro-inflammatory marker CD11b and increased expression of the anti-inflammatory cytokine IL-10 in the 4D-CTH + LSCs group compared with those in control groups (*P* < 0.05) (Fig. S4A, B, D, and E). These results suggest that 4D-CTH + LSCs not only avoid adverse immune reactions but also exert beneficial anti-inflammatory effects in the alkali-burned cornea.

### scRNA-seq analysis of porcine corneas after 7 d of 4D-CTH + LSCs treatment

Based on the results of the in vivo corneal repair experiments, the 4D-CTH + LSCs group showed markedly superior therapeutic effects compared to all other treatment groups, including the 4D-CTH group and LSCs group. Therefore, for the scRNA-seq analysis, only the 4D-CTH + LSCs group and the untreated control group were selected. This design allowed us to focus on elucidating the cellular and molecular mechanisms underlying the most effective treatment strategy, as well as to compare them directly with the pathological state of untreated alkali-burned corneas.

**Fig. 5.**
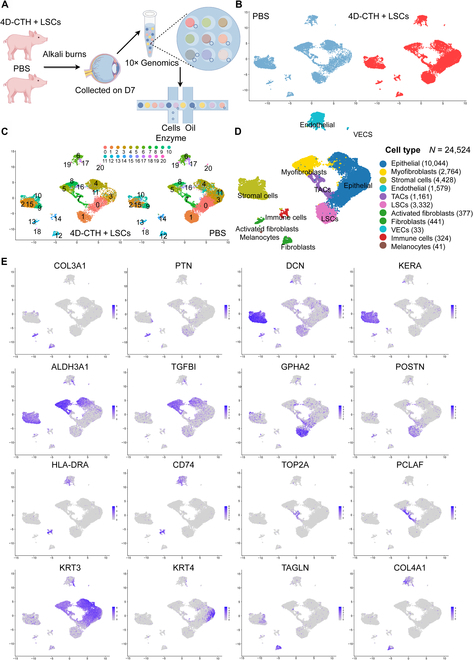
Single-cell RNA sequencing (scRNA-seq) analysis of corneal cell subtypes after alkali burn. (A) Schematic diagram of pig corneal tissue sequencing after 7 d of alkali burns. (B) Distribution of different cell populations in the corneal samples of the control group and 4D-CTH + LSCs group. (C) The control group and the 4D-CTH + LSCs group each exhibited 20 distinct cell clusters. (D) Clustering results of 24,524 cells. Different colors represent different cell populations. (E) Feather blot diagram showing the characteristic markers of various cell types. The depth of color indicates the level of expression. TACS, transport and expansion cells; VECs, vascular endothelial cells; COL3A1, collagen type III alpha 1 chain; PTN, pleiotrophin; DCN, decorin; KERA, keratocan; ALDH3A1, aldehyde dehydrogenase 3 family member A1; TGFBI, transforming growth factor beta induced; GPHA2, glycoprotein hormone subunit alpha 2; POSTN, periostin; HLA-DRA, major histocompatibility complex class II DR alpha; TOP2A, DNA topoisomerase II alpha; PCLAF, PCNA clamp associated factor; KRT3, keratin 3; KRT4, keratin 4; TAGLN, transgelin; COL4A1, collagen type IV alpha 1 chain.

To explore the molecular mechanism of 4D-CTH + LSCs in the treatment of corneal alkali burn, corneal samples from 6 young porcine pigs treated with 4D-CTH + LSCs and 6 untreated burns were collected, and single-cell samples were obtained through the 10× Genomics platform system to complete scRNA-seq analysis (Fig. 5A) [26]. All of the above cells were subjected to cluster dimensionality reduction analysis and further subcluster classification and visualization, and 20 different cell clusters were determined through the unsupervised clustering provided by the Seurat software (Fig. 5B). For the corneal samples treated with 4D-CTH + LSCs (named the treatment group), a total of 18,944 cells were sequenced, and the sequencing results of 15,327 cells were recognized to be effective. For the corneal samples of the control group (named to be untreated group during scRNA-seq analysis), a total of 10,536 cells were sequenced, and the sequencing results of 9,397 cells were recognized to be valid (Fig. 5C). Based on the differentially expressed genes of each cell subcategory and known classical markers, 11 cell types were distinguished, namely, CECs (keratin 3 and keratin 4), corneal stromal cells (decorin and keratocan), corneal endothelial cells (apolipoprotein A1 and platelet and endothelial cell adhesion molecule 1), LSCs (glycoprotein hormone subunit alpha 2 and periostin), transport and expansion cells (TACs) (DNA topoisomerase II alpha and PCNA clamp associated factor), immune cells (CD74 and major histocompatibility complex class II DR alpha), melanocytes (premelanosome protein and tyrosinase-related protein 1), activated fibroblasts (collagen type III alpha 1 chain and pleiotrophin [PTN]), fibroblasts (transgelin and collagen type IV alpha 1 chain), myofibroblasts (aldehyde dehydrogenase 3 family member A1 and transforming growth factor beta induced [TGFBI]), and vascular endothelial cells (claudin 5 and lymphatic vessel endothelial hyaluronan receptor 1) (Fig. 5D and E and Fig. S3) [27].

**Fig. 6.**
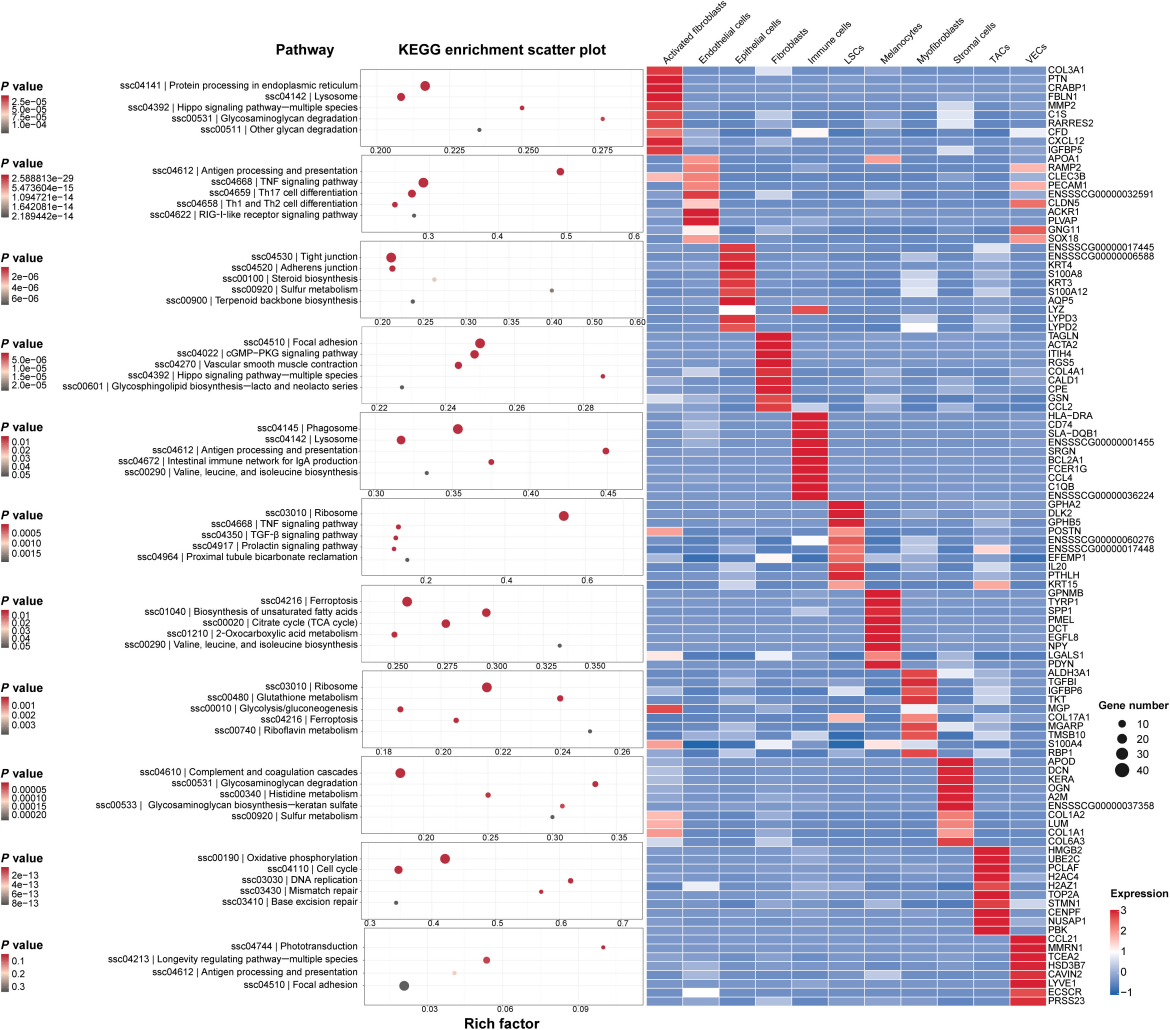
Top 10 characteristic genes of corneal cell types and related Kyoto Encyclopedia of Genes and Genomes (KEGG) pathway analysis. Expression of the top 10 differentially expressed genes (DEGs) in each cell type (right figure) and the enriched KEGG analysis pathway results (left figure). Each row represents a gene expression, each column represents a cell type, and the value of each gene is the *Z* score of the row scale.

Seven days after corneal alkali burn, different types of cells were found to exhibit different fibrosis trends. Activated fibroblasts were markedly enriched in the “ECM–receptor interaction” pathways, indicating that activated fibroblasts play a key role in ECM remodeling and fibrosis. At the same time, the “Hippo signaling pathway” regulates cell proliferation and apoptosis, promotes the activation and proliferation of fibroblasts, and promotes the fibrosis process [28]. The enrichment of the “adhesion junction” pathway of CECs reflects the re-establishment of the corneal epithelium and regulation of intercellular adhesion during the repair process, while the activity of the “keratin”-related pathway affects the structural and functional repair of epithelial cells. The “Toll-like receptor signaling pathway” of fibroblasts initiates inflammatory response and cell activation, accelerating the process of fibrosis. Immune cells participate in immune defense and tissue repair through the “Fc gamma receptor-mediated phagocytosis” and “phagosome” pathways, remove damaged cell debris, and indirectly affect the development of fibrosis. The activity of pathways such as “ribosome” and “biosynthesis of valine, leucine, and isoleucine” of LSCs provides the material and energy basis for the proliferation and differentiation of stem cells, helps tissue repair and regeneration, and may inhibit the excessive development of fibrosis. The activated “oxidative phosphorylation” and “cell cycle” pathways of myofibroblasts provide energy and cellular proliferation support for the contraction of reconstructed corneal tissue and directly participate in the fibrosis process [29]. The enrichment of pathways such as “biosynthesis of α-mannan” and “glycosaminoglycan degradation” demonstrated that stromal cells play a key role in regulating ECM metabolism and affecting tissue structure and functional remodeling. The activity of pathways such as the “oxidative phosphorylation” of transiently amplified cells ensures that sufficient cells participate in the rapid cell proliferation and repair process. Pathways such as the “glycolysis” and “starch sugar metabolism” of vascular endothelial cells involve energy metabolism and nutritional support, providing necessary conditions for tissue repair. Overall, 4D-CTH + LSCs treatment prompts the various types of cells in the newly formed corneal tissue to exhibit differential gene expression and the activation of specific signaling pathways, playing different but important roles in the re-fibrosis process of the newly formed corneal tissue after alkali burns (Fig. 6).

### Dynamic repair of alkali-burned corneas by 4D-CTH + LSCs treatment

Corneal repair is a dynamic process involving the interaction of multiple cell types [30]. LSCs and their differentiated CECs are mainly responsible for the repair and regeneration of the epithelium. Among them, LSCs with self-renewal and high proliferation potential are crucial for the healing of the corneal epithelium. Corneal stromal cells are composed of fibroblasts and myofibroblasts, which are mainly responsible for the remodeling and repair of the corneal stroma [31]. However, fibroblasts and myofibroblasts are prone to excessive proliferation when the epithelial basement membrane is damaged, leading to the formation of corneal scars [32]. Immune cells in corneal tissue may either clear pathogens and necrotic tissues through inflammatory responses or exacerbate corneal damage. The differentiation regulation of the abovementioned cells is the key to the successful corneal repair after injury.

For corneal tissues treated with 4D-CTH + LSCs, cells in the S phase (blue) and G2M phase (green) were mainly distributed in the LSC and TAC regions, indicating that 4D-CTH + LSCs treatment can promote the proliferation and differentiation of LSCs by accelerating the cell cycle. Meanwhile, the G1 phase (red) cells of the corneal tissue after 4D-CTH + LSCs treatment showed a dispersed distribution state, and some cells were undergoing a transition from the G1 phase to the S phase or G2M phase, suggesting a more diverse corneal cell differentiation state after 4D-CTH + LSCs treatment. For untreated corneal tissues, cells mostly remained in the G1 phase, while fewer were in the S phase and G2M phase, indicating that cells in the untreated group were all in a resting state and cannot provide rapid wound healing manifestations. This may be related to the impaired function or insufficient proliferation ability of stem cells. Therefore, the corneal tissue treated with 4D-CTH + LSCs showed a more active cell cycle distribution and higher stem cell proliferation than the untreated group.

**Fig. 7.**
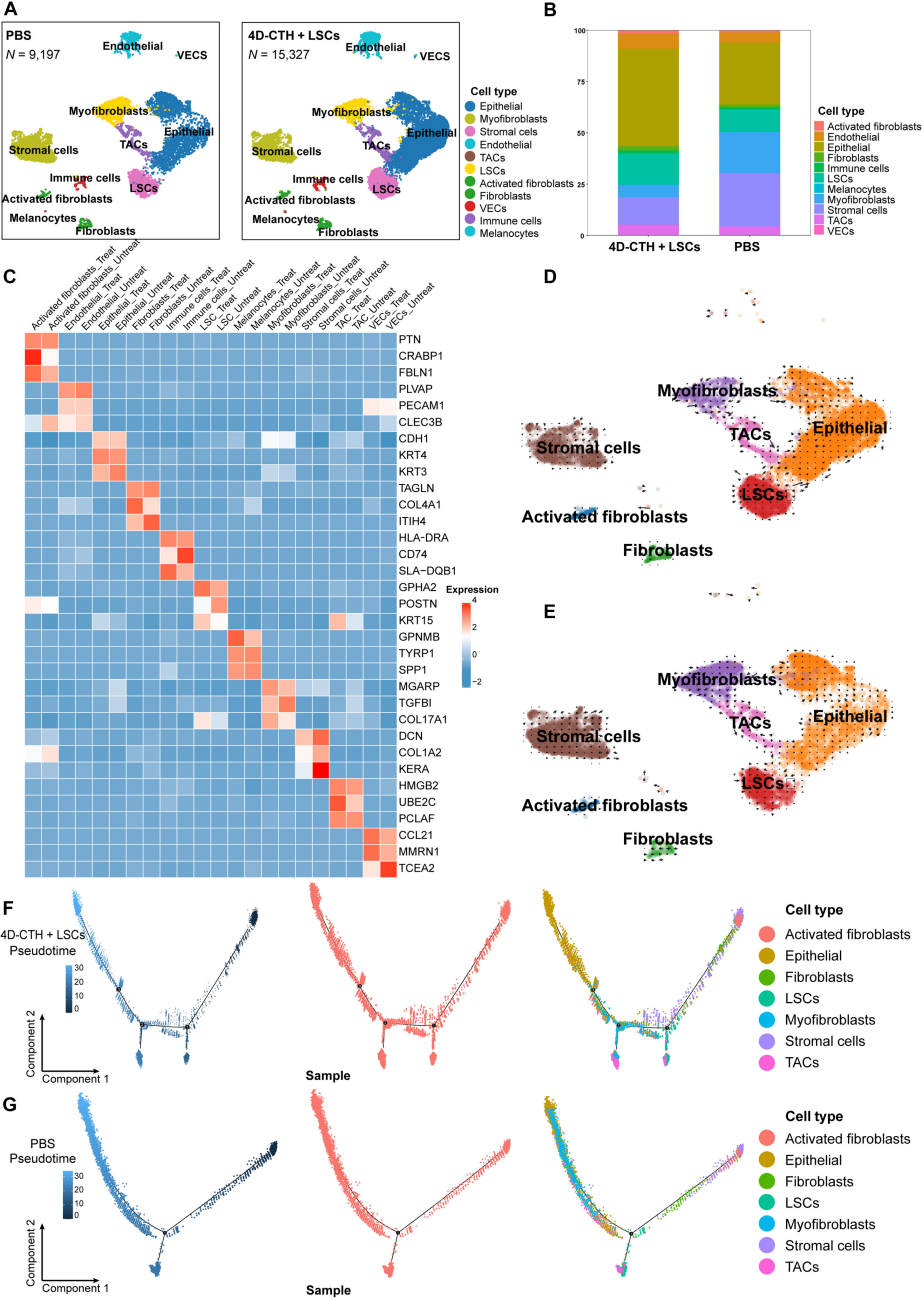
Heterogeneity and dynamic transformation of corneal cell subtypes. (A) Uniform manifold approximation and projection (UMAP) images of the 11 corneal cell subtypes in the control group and 4D-CTH + LSCs group. (B) Percentages of different cell types after annotation in the control group and 4D-CTH + LSCs group. (C) Heat map of the differential expression of classical marker genes for each cell subtype in the control group and 4D-CTH + LSCs group. (D and E) RNA rate analysis of corneal cells associated with fibrosis progression in the control group and 4D-CTH + LSCs group (dynamic model). (F and G) Projection of the pseudotemporal differentiation trajectories of corneal cells associated with fibrosis progression in the control group and 4D-CTH + LSCs group on the UMAP space. The pseudotemporal order is defined as the change from dark blue to light blue.

After 11 different types of cells were annotated, it was demonstrated that compared with the untreated group, 4D-CTH + LSCs treatment markedly down-regulated the proportion of fibroblasts and myofibroblasts and up-regulated the ratio of LSCs and CECs (Fig. 7A and B). The expressed genes of the same cell type showed notable heterogeneity in the 2 groups of samples. For example, the PTN expression of activated fibroblasts and keratin 15 expression of LSCs in the 4D-CTH + LSCs treatment group were both higher than those in the untreated group, while the TGFBI expression of myofibroblasts in the 4D-CTH + LSCs treatment group was lower than that in the untreated group (Fig. 7C). The RNA rate analysis provided by scVelo indicated that LSCs in corneal tissue exhibited a stronger tendency for myofibroblast differentiation before 4D-CTH + LSCs treatment, which further maintained corneal homeostasis by secreting antifibrotic factors or inhibiting the activation of CECs (Fig. 7E). Although this regulatory mode hindered the further development of fibrosis, it also hindered the healing of the wound surface. Correspondingly, the LSCs in the corneal tissue after 4D-CTH + LSCs treatment tended to maintain the stem cell state or differentiate to be beneficial for corneal epithelial differentiation, myofibroblast differentiation, and wound healing (Fig. 7D). The above results confirmed that 4D-CTH + LSCs can effectively inhibit and reverse fibrosis during corneal repair and promote the recovery of the normal corneal structure and function through the regulation of intercellular interactions and signal transduction.

In order to understand the pro-repair mechanism of CTS-based carriers transporting LSCs 4 d after corneal injury, the Monocle2 software was used to conduct pseudotemporal analysis on the treatment group and the untreated group, respectively, and different types of cells (epithelial cells, stromal cells, LSCs, fibroblasts, myofibroblasts, and TACs) after annotation of major categories were selected to infer the repair levels and fibrosis trends of corneal cells in the 2 groups. In the 4D-CTH + LSCs group, the transformation trajectory of cells may be more inclined to maintain or restore to a nonfibrotic state, and the distribution and transformation of myofibroblasts may be less, indicating that the 4D-CTH combined with LSCs treatment may effectively inhibit the activation and proliferation of fibrosis-related cells (such as myofibroblasts). The transformation trajectory of the cell state in the control group may show more transitions to the fibrotic state, suggesting that in the absence of therapeutic intervention, the activation and proliferation of fibrosis-related cells (such as myofibroblasts) may be more considerable (Fig. 7F and G). In the comparison of the differentiation trajectory maps of the 2 groups of cells, the nodes connected by the black lines represented the transition paths of the cell states. The gradual transformation of cells from the starting point (a darker color) to the ending point (a lighter color) indicated the changes in cell state over time or in a specific process. By comparing the cell differentiation trajectories of the 2 groups, the impact of the treatment on the cell differentiation process can be clearly observed. Among the 2 nodes presented in the control group, myofibroblasts mainly appeared in the posterior section of the second node. Among the 3 nodes in 4D-CTH + LSCs group, myofibroblasts mainly appeared near the second node, which indicated that 4D-CTH + LSCs treatment participated in the activation and differentiation of myofibroblasts. The scattered distribution of nodes and the number of branching points indicated that 4D-CTH + LSCs treatment promoted the development of cells along specific differentiation pathways and increased the diversity of cell differentiation (Fig. 7F and G). 4D-CTH + LSCs treatment may affect the transition of cell states by regulating intercellular signal transduction, thereby inhibiting fibrosis. Specifically, the earlier appearance of myofibroblasts implied that the combined treatment of 4D-CTH and LSCs promoted the early activation of these cells but subsequently inhibited their continuous activation and fibrotic effects. Conversely, in the control group, the appearance of the posterior segment of myofibroblasts might reflect the continuation of myofibroblast activation and fibrosis in the absence of therapeutic intervention of 4D-CTH + LSCs. The pseudotemporal analysis results of the 4D-CTH + LSCs treatment group and the control group suggested that 4D-CTH + LSCs treatment may promote the development of cells along specific differentiation pathways, inhibit fibrosis, and promote tissue repair and regeneration.

### 4D-CTH + LSCs treatment inhibits fibroblasts by regulating oxidative phosphorylation

**Fig. 8.**
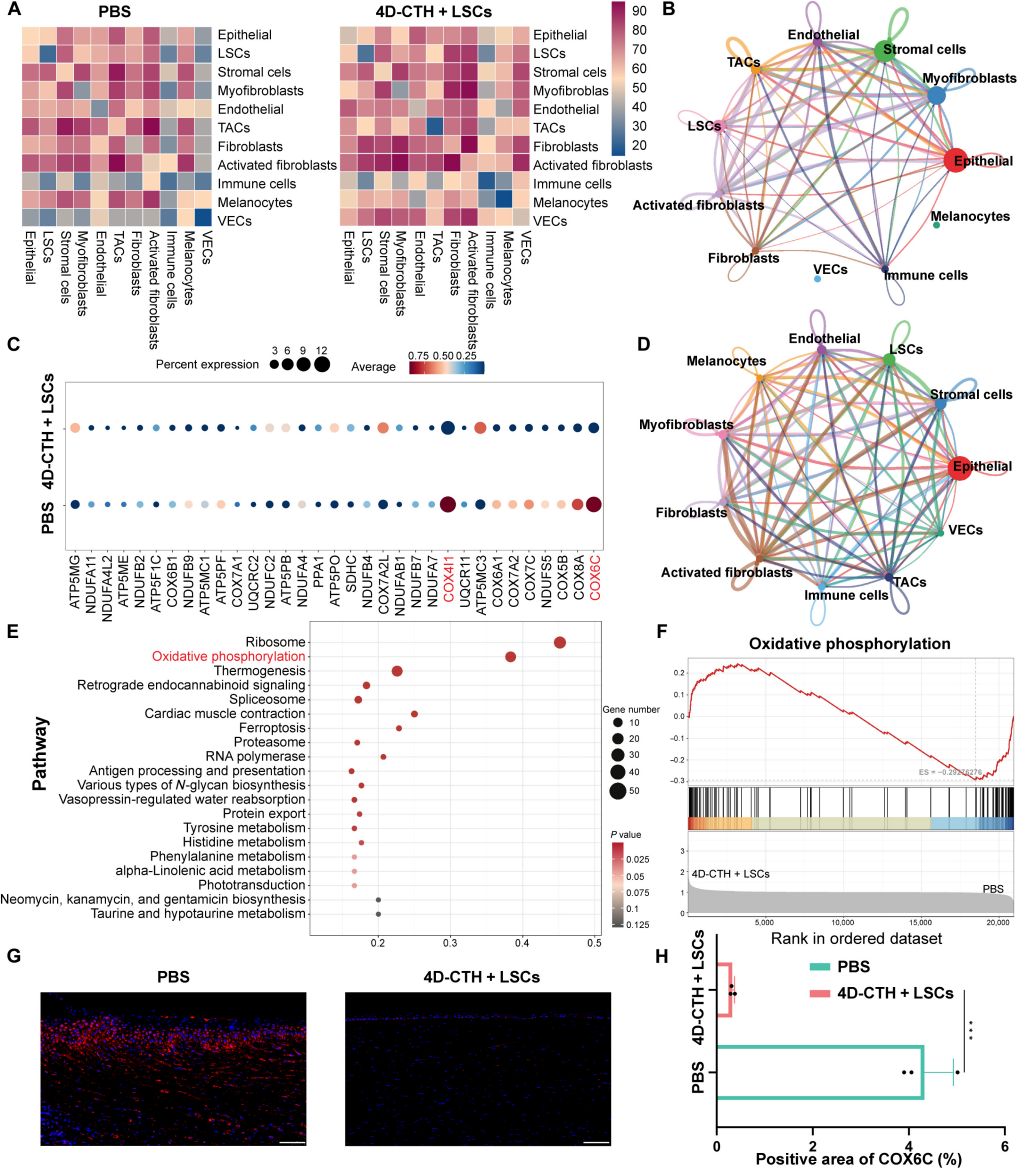
LSCs in the treatment group down-regulate cytochrome c oxidase subunit VIc (COX6C) to regulate oxidative phosphorylation. (A) Interaction heat maps of the different cell types in the control group and 4D-CTH + LSCs group, where the change in color from blue to red indicates the change in intensity from low to high. (B and D) Chord diagram of the interaction relationship of the 11 cell types in the samples of the control group and 4D-CTH + LSCs group, where the nodes represent cell types and the arcs (chords) represent the relationships between these cell types, and the thickness of the chords usually indicates the strength or quantity of the relationship. (C) Bubble chart of the average expression levels of DEGs in LSCs in the control group and 4D-CTH + LSCs group. The depth of the color corresponds to the high and low expression levels of the gene. (E) KEGG pathway analysis of DEGs in LSCs. (F) Gene enrichment analysis results, showing the enrichment score (ES) for each gene set. (G) Fluorescence staining images of COX6C in samples of the control group and 4D-CTH + LSCs group; scale bar: 200 μm. (H) Quantitative analysis of COX6C expression in the control group and 4D-CTH + LSCs group. ***P < 0.001.

4D-CTH + LSCs-treated corneal tissues and untreated corneal tissues were subjected to pseudotime analysis and RNA rate analysis, which confirmed that 4D-CTH + LSCs treatment may inhibit fibrosis progression by regulating the differentiation and activation state of fibrosis-related cells. In order to further explore the mechanism by which 4D-CTH + LSCs affects corneal repair, 4D-CTH + LSCs-treated corneal tissues and untreated corneal tissues were subjected to cell communication analysis. Specifically, CellPhoneDB analysis evaluated the interaction strength between different cell types. Darker colors in the heat map of the cell-to-cell communication network visualization correspond to higher communication probabilities. 4D-CTH + LSCs-treated corneal tissues showed enhanced communication between LSCs and fibrosis-related cells (fibroblasts), suggesting that tissue repair promoted by 4D-CTH + LSCs treatment is closely related to fibrosis control (Fig. 8A). In addition, the corneal tissue treated with 4D-CTH + LSCs showed stronger interactions between LSCs and CECs, which was consistent with faster corneal epithelial repair (Fig. 8A). The chord diagram displayed by CellChat analysis showed a more active intercellular communication network in the 4D-CTH + LSCs-treated corneal tissue, which is of great significance for faster epithelialization and orderly corneal tissue reconstruction (Fig. 8B and D).

The results of differential gene enrichment analysis of LSCs showed that the genes with notable differences in expression between the control group and the 4D-CTH + LSCs group were mainly related to mitochondrial function. Specifically, the expression of COX6C and COX4I1 genes (genes encoding complex IV of the mitochondrial respiratory chain) after 4D-CTH + LSCs treatment was markedly lower than those in the untreated group (Fig. 8C). KEGG analysis further confirmed that the above differentially expressed genes were mainly involved in the oxidative-phosphorylation-related pathway, which was markedly regulated by COX6C (Fig. 8E) [33]. The above results suggested that 4D-CTH + LSCs treatment may inhibit oxidative phosphorylation by reducing the expression of the COX6C gene, thereby inhibiting fibrosis. GSEA confirmed the above conclusions. Specifically, GSEA first confirmed the significantly up-regulated oxidative phosphorylation and the up-regulated expression of multiple related genes in the untreated group (Fig. 8F). The results of COX6C fluorescence staining of corneal tissues treated for 7 d proved that the expression of COX6C in the 4D-CTH + LSCs treatment group was significantly lower than that of the untreated group (*P* < 0.05, *) (Fig. 8G and H). In summary, 4D-CTH + LSCs treatment was proven to down-regulate COX6C to inhibit oxidative phosphorylation of burned corneal cells and regulate the expression of fibrosis-related cells (fibroblasts), thereby inhibiting the fibrosis of new corneal tissues.

## Discussion

Severe corneal injury often leads to LSCD, which is clinically manifested as inward growth of the conjunctiva and corneal epithelium, vascularization, chronic inflammation, repeated erosion, persistent ulcers, destruction of the basement membrane, and inward growth of fibrous tissue. Ultimately, it leads to severe impairment of corneal function, interruption of CEC renewal, and decline in vision [34]. Human corneal transplantation is currently the most effective strategy for the clinical treatment of LSCD, but it requires healthy implants, advanced surgical techniques, and professional equipment and is markedly constrained by the severe shortage of donors [35]. In addition, the risks of immune rejection and infection hinder the promotion of allogeneic grafts, especially in patients with inflammation and severe corneal lesions. At present, human amniotic membrane with anti-inflammatory, antibacterial, and antiangiogenic properties is the most commonly used stent for the treatment of ocular surface diseases. However, fatal drawbacks such as expensive donor screening, low transparency, poor tensile strength, large batch differences, and poor prognosis caused by postoperative rejection reactions have also hindered the clinical promotion of human amniotic membrane [36].

The clinical treatment of patients with severe corneal injury is limited not only by the lack of donor corneas but also by the unsuccessful explanation of the promotion of corneal epithelial integrity by donor cells [37]. Therefore, the exploration of the healing mechanism of corneal tissue is crucial for the improvement of treatment strategies for severe corneal injury. On the one hand, the healing of corneal wounds involves the restoration of the niche environment composed of numerous cells, including LSCs, CECs, immune cells, stromal cells, endothelial cells, and ECM. On the other hand, the limbal niche needs to provide a precise and orderly 3D spatial environment for numerous cellular members, including LSCs, to maintain the special structure of the cornea and its complex visual functions.

The development of satisfactory tissue engineering carriers relies on the preparation and optimization of hydrogel materials with high biosafety, including the optimization of components and preparation processes [38]. Tissue engineering carriers provide a favorable growth support environment for cells and are widely used in many fields, including disease modeling, regenerative medicine, drug testing, personalized medicine, organ development, toxicity research, and implants [39]. Unfortunately, traditional tissue engineering carriers, due to the low internal space porosity, uneven pore diameters, and lack of precise regulation, are currently unable to provide a satisfactory microenvironment for seed cells in alkali burn treatment [40].

CTS, as a nontoxic and bioabsorbable natural polysaccharide, is an ideal candidate for the carrier of tissue engineering hydrogels [41]. The excellent biocompatibility, antibacterial properties, and mucosal adhesion characteristics make CTS a potential degradable carrier scaffold in corneal wound healing applications [42]. Compared with amniotic membranes, tissue-engineered CTS hydrogels exhibits considerable advantages including high transparency, a uniform structure, controllable physical properties, and low rejection reactions [43]. CTS hydrogels do not require extensive serological screening before clinical application, avoiding complex preoperative operations. In addition, the internal network structure of CTS hydrogel is also suitable for the intervention and guidance of stem cell fate [44].

“4D-bioprinting technology”, which is developed on the basis of 3D bioprinting, utilizes biomaterials with shape-memory functions and 3D-bioprinting strategies to automatically print the required shapes and pore sizes under different stimulation conditions such as light, heat, pH, and magnetic stimulation [16]. 4D-bioprinting technology can provide biological carriers with multiscale micro–nano hierarchical pore structures that change shape or behavior over time [12]. After the seed cells (stem cells) loaded by 4D-bioprinting hydrogels are transported to the surface of the damaged tissue, the hydrogel is gradually degraded and absorbed by the host tissue, while the implanted cells participate in the tissue repair through proliferation and differentiation. Therefore, the 4D-bioprinting CTS-based hydrogel is expected to provide a biomimetic ECM environment closer to that of the human body and promote the differentiation of stem cells for the rapid repair of injured tissues [17]. Although the current study demonstrated the biocompatibility and anti-inflammatory effects of the 4D-CTH hydrogel, the surface topography, especially the roughness of the scaffold, was not quantitatively analyzed. Surface roughness is known to influence cell adhesion, proliferation, and migration by affecting cell–material interactions at the micro- and nanoscale [45]. Given its potential significance, future work should include detailed characterization of surface roughness using techniques such as atomic force microscopy or 3D profilometry, to better understand its role in modulating cellular responses and improving scaffold performance. Although the abovementioned innovative achievements were obtained in this study, the promotion of this technology in the treatment of LSCD still faces the following challenges: First of all, given that 4D-CTH + LSCs treatment was used only in a large-animal LSCD model in this study, other animal models, such as rhesus monkeys, should be used to verify the effectiveness of 4D-CTH + LSCs treatment in the future. Second, more convincing research conclusions on 4D-CTH + LSCs treatment still need to be supported by a large amount of clinical sample data.

In this study, an EGF-loaded hydrogel, a clinically approved and widely used treatment for ocular surface injuries, was selected as the positive control group. This design is beneficial for the evaluation of the therapeutic performance of the 4D-CTH + LSCs system according to the clinical standard. Therefore, the above comparison of 4D-CTH + LSCs with EGF-loaded hydrogel could reflect the clinical advantages or translational value of the proposed approach. In addition, several critical issues should be addressed before clinical translation. The scalability of 4D-printed LSCs constructs remains uncertain, as large-scale manufacturing while maintaining the structural fidelity and biological activity of stem cells is technically challenging. The sterilization of hydrogel-based bioprinted carriers is another hurdle, since conventional high-temperature or irradiation sterilization may alter the physicochemical properties of CTS hydrogels and compromise cell viability, necessitating the development of gentle yet effective sterilization methods [46]. Furthermore, the storage and transportation of living-cell-loaded constructs require stringent temperature control and preservation conditions, which may limit their application in resource-limited clinical settings. From a regulatory perspective, the approval pathway for combined products containing both living cells and 4D-printed biomaterials is complex, involving multiple evaluations for safety, efficacy, and manufacturing consistency [47].

Beyond the above challenges, manufacturing consistency must be ensured, as variations in printing parameters, mechanical properties, or cell distribution between batches could affect therapeutic outcomes. The cost-effectiveness of this technology should also be considered, since high-precision 4D-bioprinting equipment, materials, and cell culture processes may be economically challenging for routine clinical use [48]. Moreover, despite the autologous nature of LSCs, potential immunogenicity and biosafety issues related to hydrogel degradation products and residual printing chemicals require long-term safety assessments. Finally, the long-term efficacy and functional restoration of corneal transparency and visual quality, as well as the impact of patient heterogeneity on therapeutic response, must be validated in large-scale, multicenter, and well-controlled clinical trials before 4D-CTH + LSCs can be widely applied in clinical practice.

In this work, 4D-bioprinting technology was applied to prepare CTS-based tissue-engineered hydrogel carriers (4D-CTH) with high porosity, a uniform pore size, adjustable morphology, and interconnected pores. Specifically, the antibacterial performance and its improvement of 4D-CTH in terms of the proliferation and migration of LSCs were verified. MTT assays demonstrated that the scaffold exhibited good cytocompatibility with LSCs. In vitro degradation tests confirmed the satisfactory biodegradability of the 4D-CTH scaffold. In vivo evaluation indicated that LSCs loaded onto the 4D-CTH scaffold could markedly accelerate the wound repair process, which was further confirmed by the results of HE staining, Masson staining, and Ki67 and α-SMA immunohistochemical staining. Furthermore, the synergistic effect of 4D-CTH and LSCs can effectively alleviate the inflammatory response, which may be attributed to the suitable microenvironment provided by 4D-CTH for the growth and proliferation of LSCs and the reduced infiltration of inflammatory cells. Immunohistochemical staining of corneal sections on D7 post-alkali burn further revealed decreased CD11b expression and increased IL-10 expression in the 4D-CTH + LSCs group, confirming its excellent anti-inflammatory properties. Additionally, Alcian Blue staining again indicated the absence of residual CTS in the corneal epithelium. Additionally, single-cell sequencing technology combined with bioinformatics was adopted for systematic research. The results confirmed that 4D-CTH can substantially increase the proportion of LSCs in corneal tissue by promoting the residence and growth of LSCs. Additionally, 4D-CTH loaded with LSCs can inhibit and reverse corneal fibrosis by interfering with fibroblast differentiation, which is closely related to the down-regulation of COX6C expression by LSCs, thereby inhibiting oxidative phosphorylation in fibroblasts. Therefore, based on the demonstration of the feasibility of 4D-CTH + LSCs for the treatment of corneal burned by alkali, this work clarified the regulation mechanism of corneal epithelial homeostasis by 4D-CTH + LSCs, providing theoretical support and an application paradigm for corneal tissue engineering therapy.

## Data Availability

The datasets used and/or analyzed in this study are available from the corresponding authors on reasonable request.

## References

[1] Sun X, Song W, Teng L, Huang Y, Liu J, Peng Y, Lu X, Yuan J, Zhao X, Zhao Q. MiRNA 24-3p-rich exosomes functionalized DEGMA-modified hyaluronic acid hydrogels for corneal epithelial healing. Bioact Mater. 2023;25:640–656. 37056274 10.1016/j.bioactmat.2022.07.011 PMC10086767

[2] Duan F, Chen X, Zhang S, Qi X, Shi W, Gao H. Clinical characteristics and visual outcomes of patients hospitalized for ocular trauma in Shandong Province, China. J Ophthalmol. 2020;2020:5826263. 32377421 10.1155/2020/5826263 PMC7180499

[3] Villabona-Martinez V, Sampaio LP, Shiju TM, Wilson SE. Standardization of corneal alkali burn methodology in rabbits. Exp Eye Res. 2023;230: Article 109443. 36948438 10.1016/j.exer.2023.109443

[4] Shi X, Zhou T, Huang S, Yao Y, Xu P, Hu S, Tu C, Yin W, Gao C, Ye J. An electrospun scaffold functionalized with a ROS-scavenging hydrogel stimulates ocular wound healing. Acta Biomater. 2023;158:266–280. 36638943 10.1016/j.actbio.2023.01.016

[5] Soleimani M, Mirshahi R, Cheraqpour K, Baharnoori SM, Massoumi H, Chow C, Shahjahan S, Momenaei B, Ashraf MJ, Koganti R, et al. Intrastromal versus subconjunctival injection of mesenchymal stem/stromal cells for promoting corneal repair. Ocul Surf. 2023;30:187–195. 37758115 10.1016/j.jtos.2023.09.008 PMC10841412

[6] Zekušić M, Bujić Mihica M, Skoko M, Vukušić K, Risteski P, Martinčić J, Tolić IM, Bendelja K, Ramić S, Dolenec T, et al. New characterization and safety evaluation of human limbal stem cells used in clinical application: Fidelity of mitotic process and mitotic spindle morphologies. Stem Cell Res Ther. 2023;14(1):368. 38093301 10.1186/s13287-023-03586-z PMC10720168

[7] Masood F, Chang JH, Akbar A, Song A, Hu WY, Azar DT, Rosenblatt MI. Therapeutic strategies for restoring perturbed corneal epithelial homeostasis in limbal stem cell deficiency: Current trends and future directions. Cells. 2022;11(20):3247. 36291115 10.3390/cells11203247 PMC9600167

[8] Tonti E, Manco GA, Spadea L, Zeppieri M. Focus on limbal stem cell deficiency and limbal cell transplantation. World J Transplant. 2023;13(6):321–330. 38174150 10.5500/wjt.v13.i6.321 PMC10758683

[9] Elhusseiny AM, Soleimani M, Eleiwa TK, ElSheikh RH, Frank CR, Naderan M, Yazdanpanah G, Rosenblatt MI, Djalilian AR. Current and emerging therapies for limbal stem cell deficiency. Stem Cells Transl Med. 2022;11(3):259–268. 35303110 10.1093/stcltm/szab028 PMC8968724

[10] Norrick A, Esterlechner J, Niebergall-Roth E, Dehio U, Sadeghi S, Schröder HM, Ballikaya S, Stemler N, Ganss C, Dieter K, et al. Process development and safety evaluation of ABCB5^+^ limbal stem cells as advanced-therapy medicinal product to treat limbal stem cell deficiency. Stem Cell Res Ther. 2021;12(1):194. 33741066 10.1186/s13287-021-02272-2 PMC7980611

[11] Robbins BT, Montreuil KA, Kundu N, Kumar P, Agrahari V. Corneal treatment, repair, and regeneration: Exosomes at rescue. Pharmaceutics. 2024;16(11):1424. 39598547 10.3390/pharmaceutics16111424 PMC11597686

[12] Zhuo S, Geever LM, Halligan E, Tie BSH, Breheny C. A development of new material for 4D printing and the material properties comparison between the conventional and stereolithography polymerised NVCL hydrogels. J Funct Biomater. 2022;13(4):262. 36547522 10.3390/jfb13040262 PMC9785372

[13] Agarwal T, Chiesa I, Costantini M, Lopamarda A, Tirelli MC, Borra OP, Varshapally SVS, Kumar YAV, Koteswara Reddy G, De Maria C, et al. Chitosan and its derivatives in 3D/4D (bio) printing for tissue engineering and drug delivery applications. Int J Biol Macromol. 2023;246: Article 125669. 37406901 10.1016/j.ijbiomac.2023.125669

[14] Li Y, Wang Z. Biomaterials for corneal regeneration. Adv Sci. 2025;12(6): Article e2408021. 10.1002/advs.202408021 PMC11809424 39739318

[15] Faber L, Yau A, Chen Y. Translational biomaterials of four-dimensional bioprinting for tissue regeneration. Biofabrication. 2023;16(1): Article 012001. 10.1088/1758-5090/acfdd0 PMC10561158 37757814

[16] Yarali E, Mirzaali MJ, Ghalayaniesfahani A, Accardo A, Diaz-Payno PJ, Zadpoor AA. 4D printing for biomedical applications. Adv Mater. 2024;36(31): Article e2402301. 38580291 10.1002/adma.202402301

[17] Lai J, Liu Y, Lu G, Yung P, Wang X, Tuan RS, Li ZA. 4D bioprinting of programmed dynamic tissues. Bioact Mater. 2024;37:348–377. 38694766 10.1016/j.bioactmat.2024.03.033 PMC11061618

[18] Ding A, Lee SJ, Tang R, Gasvoda KL, He F, Alsberg E. 4D cell-condensate bioprinting. Small. 2022;18(36): Article e2202196. 35973946 10.1002/smll.202202196 PMC9463124

[19] Wang Z, Jiang C, Fan Y, Hao X, Dong Y, He X, Gao J, Zhang Y, Li M, Wang M, et al. The application of a 4D-printed chitosan-based stem cell carrier for the repair of corneal alkali burns. Stem Cell Res Ther. 2024;15(1):41. 38355568 10.1186/s13287-024-03653-z PMC10865625

[20] Su G, Guo X, Xu L, Jin B, Tan Y, Zhou X, Wang W, Li X, Wang S, Li G. Isolation and characterization of rabbit limbal niche cells. Exp Eye Res. 2024;241: Article 109838. 38395213 10.1016/j.exer.2024.109838

[21] Nakamichi A, Kadokawa JI. Fabrication of chitosan-based network polysaccharide nanogels. Molecules. 2022;27(23):8384. 36500476 10.3390/molecules27238384 PMC9740819

[22] Xie HT, Chen SY, Li GG, Tseng SCG. Limbal epithelial stem/progenitor cells attract stromal niche cells by SDF-1/CXCR4 signaling to prevent differentiation. Stem Cells. 2011;29(11):1874–1885. 21948620 10.1002/stem.743

[23] Ghoubay-Benallaoua D, de Sousa C, Martos R, Latour G, Schanne-Klein MC, Dupin E, Borderie V. Easy xeno-free and feeder-free method for isolating and growing limbal stromal and epithelial stem cells of the human cornea. PLOS ONE. 2017;12(11): Article e0188398. 29149196 10.1371/journal.pone.0188398 PMC5693460

[24] Prusek A, Sikora B, Skubis-Sikora A, Czekaj P. Assessment of the toxic effect of benzalkonium chloride on human limbal stem cells. Sci Rep. 2025;15(1):12295. 40210649 10.1038/s41598-025-96919-2 PMC11986071

[25] Xie ZJ, Yuan BW, Chi MM, Hong J. Focus on seed cells: Stem cells in 3D bioprinting of corneal grafts. Front Bioeng Biotechnol. 2024;12:1423864. 39050685 10.3389/fbioe.2024.1423864 PMC11267584

[26] Zhou M, Shi ZX, Liu Z, Ke SR, Wang CY, Liang XL, Hu QL, Zhang QK, Wang DL, Sun L, et al. Single-cell transcriptomic analysis reveals dynamic cellular processes in corneal epithelium during wound healing in cynomolgus monkeys. Invest Ophthalmol Vis Sci. 2024;65(11):43. 10.1167/iovs.65.11.43 PMC11437678 39330987

[27] Sun D, Zhang X, Chen R, Sang T, Li Y, Wang Q, Xie L, Zhou Q, Dou S. Decoding cellular plasticity and niche regulation of limbal stem cells during corneal wound healing. Stem Cell Res Ther. 2024;15(1):201. 38971839 10.1186/s13287-024-03816-y PMC11227725

[28] Gokey JJ, Patel SD, Kropski JA. The role of Hippo/YAP signaling in alveolar repair and pulmonary fibrosis. Front Med. 2021;8: Article 752316. 10.3389/fmed.2021.752316 PMC8520933 34671628

[29] Wilson SE. Corneal myofibroblasts and fibrosis. Exp Eye Res. 2020;201: Article 108272. 33010289 10.1016/j.exer.2020.108272 PMC7736212

[30] Shadmani A, Wu AY. Navigating the path to corneal healing success and challenges: A comprehensive overview. Eye. 2025;39(6):1047–1055. 39939391 10.1038/s41433-025-03619-2 PMC11978883

[31] Kumar A, Yun H, Funderburgh ML, Du Y. Regenerative therapy for the cornea. Prog Retin Eye Res. 2022;87: Article 101011. 34530154 10.1016/j.preteyeres.2021.101011 PMC8918435

[32] Jeon KI, Kumar A, Brookes PS, Nehrke K, Huxlin KR. Manipulating mitochondrial pyruvate carrier function causes metabolic remodeling in corneal myofibroblasts that ameliorates fibrosis. Redox Biol. 2024;75: Article 103235. 38889622 10.1016/j.redox.2024.103235 PMC11231598

[33] Liu S, Shao F, Wang Y, Zhang Y, Yu H, Zhang N, He L, Kong Q, Jiang H, Dong Z. COX6C expression driven by copy amplification of 8q22.2 regulates cell proliferation via mediation of mitosis by ROS-AMPK signaling in lung adenocarcinoma. Cell Death Dis. 2024;15(1):74. 38242874 10.1038/s41419-024-06443-w PMC10799076

[34] Kethiri AR, Raju E, Bokara KK, Mishra DK, Basu S, Rao CM, Sangwan VS, Singh V. Inflammation, vascularization and goblet cell differences in LSCD: Validating animal models of corneal alkali burns. Exp Eye Res. 2019;185: Article 107665. 31095932 10.1016/j.exer.2019.05.005

[35] Islam MM, Buznyk O, Reddy JC, Pasyechnikova N, Alarcon EI, Hayes S, Lewis P, Fagerholm P, He C, Iakymenko S, et al. Biomaterials-enabled cornea regeneration in patients at high risk for rejection of donor tissue transplantation. NPJ Regen Med. 2018;3:2. 29423280 10.1038/s41536-017-0038-8 PMC5792605

[36] Ramuta TŽ, Šket T, Starčič Erjavec M, Kreft ME. Antimicrobial activity of human fetal membranes: From biological function to clinical use. Front Bioeng Biotechnol. 2021;9: Article 691522. 34136474 10.3389/fbioe.2021.691522 PMC8201995

[37] Zhang K, Guo MY, Li QG, Wang XH, Wan YY, Yang ZJ, He M, Yi YM, Jiang LP, Qu XH, et al. Drp1-dependent mitochondrial fission mediates corneal injury induced by alkali burn. Free Radic Biol Med. 2021;176:149–161. 34562609 10.1016/j.freeradbiomed.2021.09.019

[38] Zhong Y, Seidi F, Wang Y, Zheng L, Jin Y, Xiao H. Injectable chitosan hydrogels tailored with antibacterial and antioxidant dual functions for regenerative wound healing. Carbohydr Polym. 2022;298: Article 120103. 36241280 10.1016/j.carbpol.2022.120103

[39] Balters L, Reichl S. 3D bioprinting of corneal models: A review of the current state and future outlook. J Tissue Eng. 2023;14:20417314231197793. 37719307 10.1177/20417314231197793 PMC10504850

[40] Singh V, Tiwari A, Kethiri AR, Sangwan VS. Current perspectives of limbal-derived stem cells and its application in ocular surface regeneration and limbal stem cell transplantation. Stem Cells Transl Med. 2021;10(8):1121–1128. 33951336 10.1002/sctm.20-0408 PMC8284782

[41] Peng W, Li D, Dai K, Wang Y, Song P, Li H, Tang P, Zhang Z, Li Z, Zhou Y, et al. Recent progress of collagen, chitosan, alginate and other hydrogels in skin repair and wound dressing applications. Int J Biol Macromol. 2022;208:400–408. 35248609 10.1016/j.ijbiomac.2022.03.002

[42] Deng P, Yao L, Chen J, Tang Z, Zhou J. Chitosan-based hydrogels with injectable, self-healing and antibacterial properties for wound healing. Carbohydr Polym. 2022;276: Article 118718. 34823762 10.1016/j.carbpol.2021.118718

[43] Bakhshandeh H, Atyabi F, Soleimani M, Taherzadeh ES, Shahhoseini S, Cohan RA. Biocompatibility improvement of artificial cornea using chitosan-dextran nanoparticles containing bioactive macromolecules obtained from human amniotic membrane. Int J Biol Macromol. 2021;169:492–499. 33358948 10.1016/j.ijbiomac.2020.12.125

[44] Gwon K, Hong HJ, Gonzalez-Suarez AM, Slama MQ, Choi D, Hong J, Baskaran H, Stybayeva G, Peterson QP, Revzin A. Bioactive hydrogel microcapsules for guiding stem cell fate decisions by release and reloading of growth factors. Bioact Mater. 2022;15:1–14. 35386345 10.1016/j.bioactmat.2021.12.008 PMC8941170

[45] Noroozi R, Arif ZU, Taghvaei H, Khalid MY, Sahbafar H, Hadi A, Sadeghianmaryan A, Chen X. 3D and 4D bioprinting technologies: A game changer for the biomedical sector? Ann Biomed Eng. 2023;51(8):1683–1712. 37261588 10.1007/s10439-023-03243-9

[46] Garvey M. Medical device-associated healthcare infections: Sterilization and the potential of novel biological approaches to ensure patient safety. Int J Mol Sci. 2023;25(1):201. 38203372 10.3390/ijms25010201 PMC10778788

[47] Dey M, Ozbolat IT. 3D bioprinting of cells, tissues and organs. Sci Rep. 2020;10(1):14023. 32811864 10.1038/s41598-020-70086-y PMC7434768

[48] Beetler DJ, Di Florio DN, Law EW, Groen CM, Windebank AJ, Peterson QP, Fairweather D. The evolving regulatory landscape in regenerative medicine. Mol Asp Med. 2023;91: Article 101138. 10.1016/j.mam.2022.101138 PMC10162454 36050142

