# Supplementary material for: Repairing Effect and Mechanism of the 4-Dimensionally Printed Limbal Stem Cell Strategy on Corneal Alkali Burns in Large Animals
Source: Biomater Res. 2025 Oct 2;29:0262. doi: 10.34133/bmr.0262 (PMC12489182; doi:10.34133/bmr.0262)
Supplement: Supplementary 1 — Figs. S1 to S4 [file bmr.0262.f1.docx]

**Supplementary Information**

**The following supplementary files are provided to support the findings reported in the main manuscript:**

**Supplementary Figure S1:** In vitro characterization of 4D-printed hydrogels, including MTT cytotoxicity assessment and degradation profiles. These data complement Figure 1 in the main text.

**Supplementary Figure S2:** Application of 4D-CTH hydrogels in the corneal alkali burn model. These images provide additional details on the treatment outcomes, complementing Figures 2 in the main text.

**Supplementary Figure S3:** Evaluation of organ toxicity. Histological analyses are presented to assess systemic safety.

**Supplementary Figure S4:** Inflammatory marker analysis and Alcian Blue staining of chitosan. These data provide further insights into inflammation regulation and chitosan distribution in the hydrogels, complementing the main results.

**
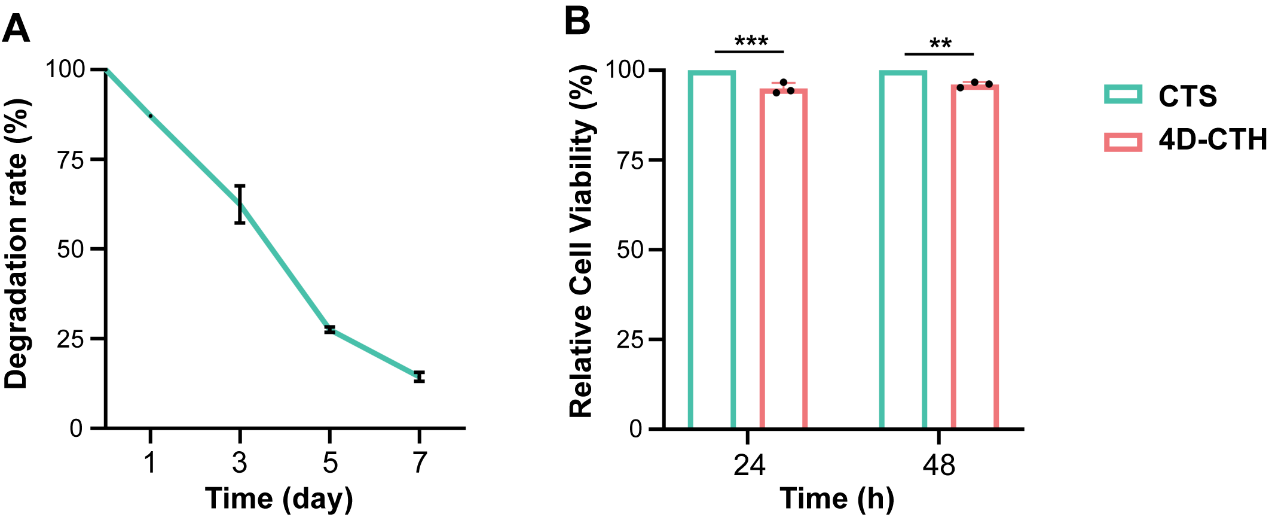
**

**Supplementary Figure 1 *In vitro* degradation of chitosan and cytocompatibility evaluation by MTT assay.** **A:** Quantitative analysis of the *in vitro* degradation rate of chitosan over time. **B:** Cell viability (%) assessed by MTT assay after exposure to different concentrations of chitosan extract. Data are presented as mean ± SD (n = 3 independent experiments). ***P* < 0.01, ****P* < 0.001, *****P* < 0.0001 versus control.


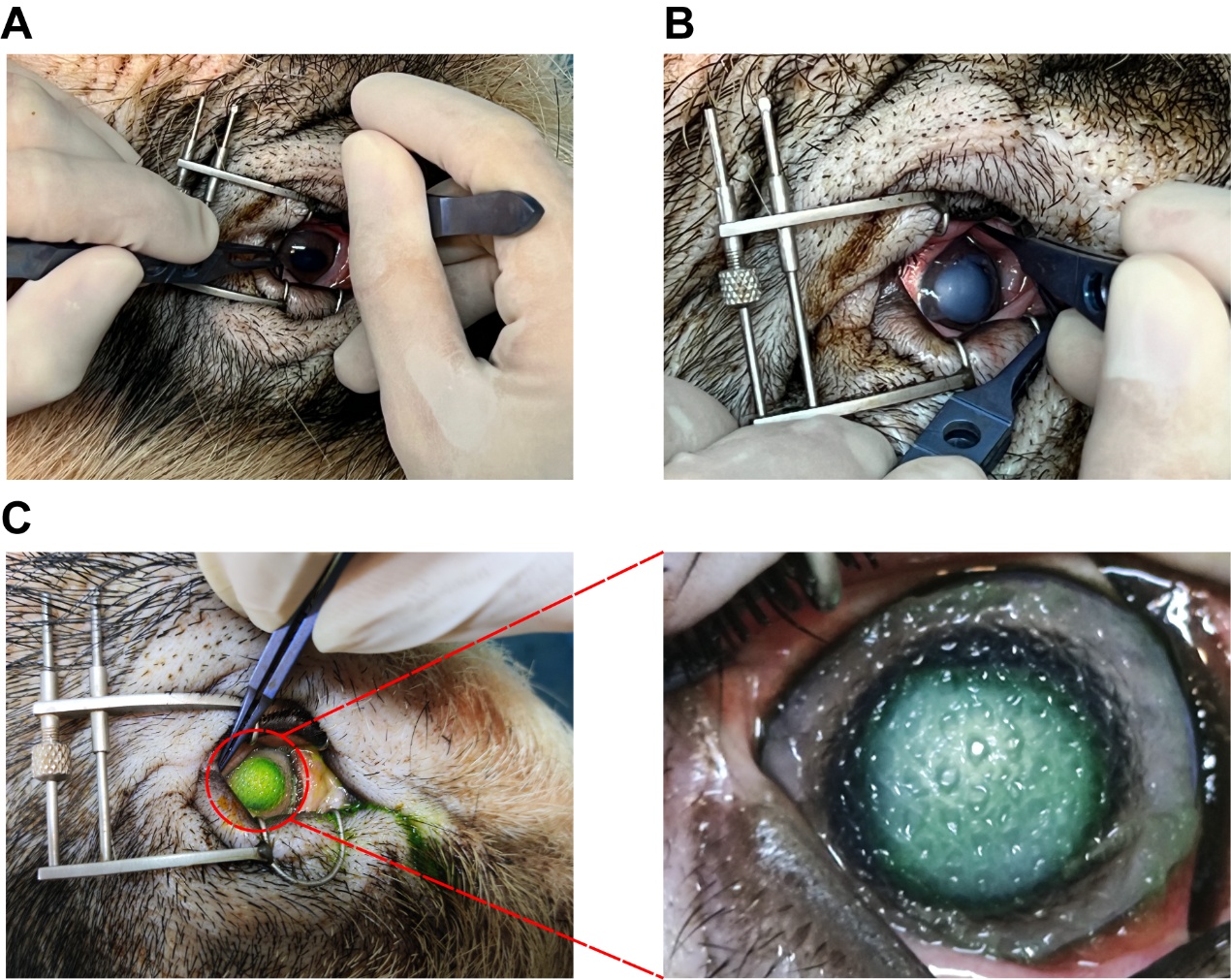


**Supplementary Figure 2. Construction of corneal alkali burn model**. **A:** Preparation before establishing the corneal alkali burn model of the right eye of the miniature pig. **B:** Image of the corneal damaged area after alkali burn. **C:** Image of 4D-CTH attached to the burned cornea area.


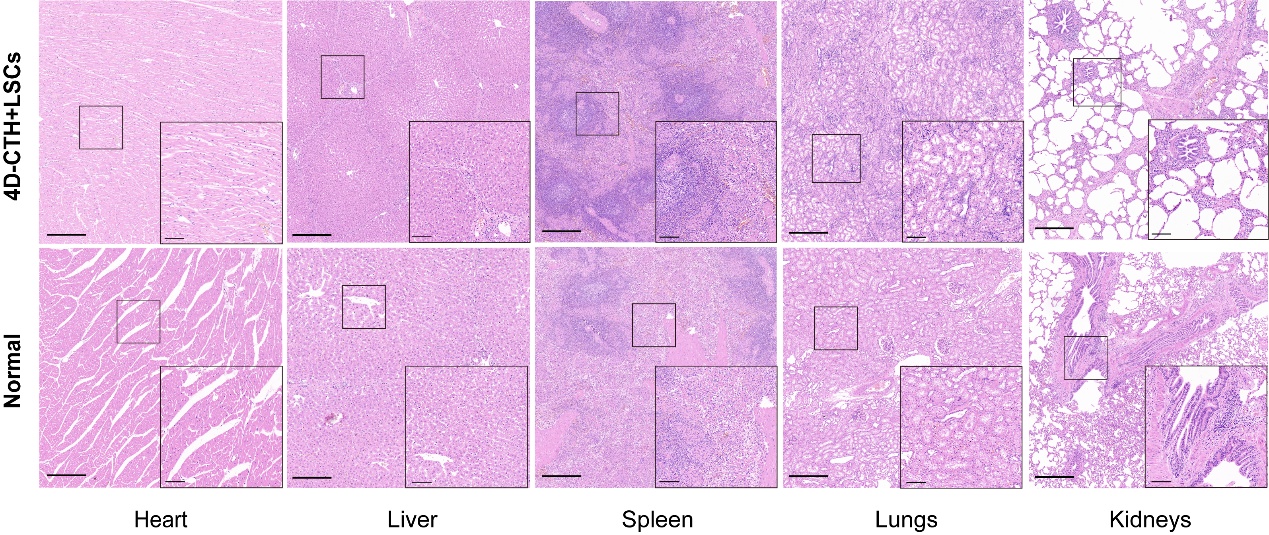


**Supplementary Figure 3. Organ toxicity of 4D-CTH** Effects of 4D-CTH+LSCs group on heart, liver, spleen, lung and kidney tissues. Scale bar, 200 μm.


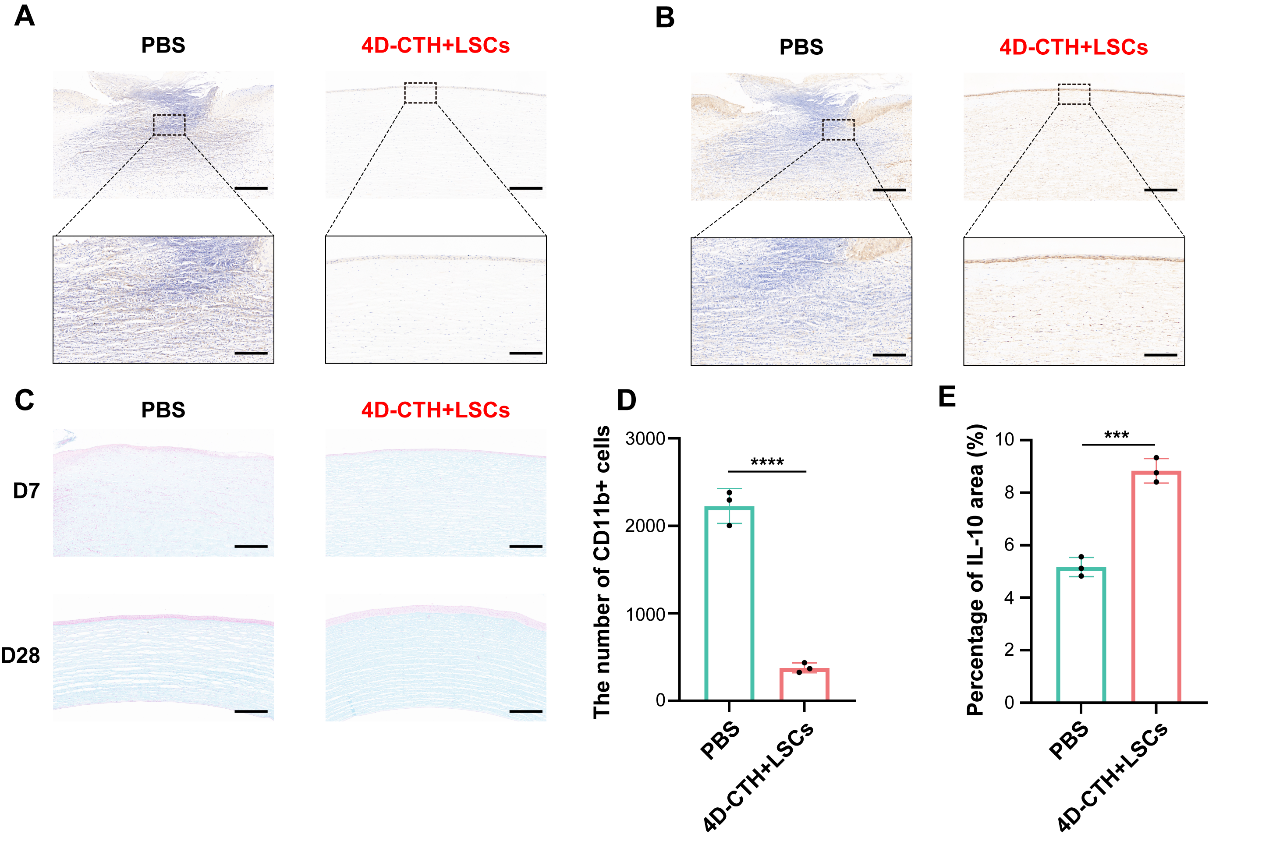


**Supplementary Figure4. Evaluation of inflammatory response and *in vivo* degradation of 4D-CTH after transplantation. A**: Representative images of CD11b immunohistochemical staining in injured corneas from PBS and 4D-CTH+LSCs groups at day 7 (D7). **B**: Representative images of IL-10 immunohistochemical staining in corneal tissues from both groups at day 7 (D7). **C**: Alcian blue staining to evaluate in vivo residual chitosan in transplanted 4D-CTH scaffolds at day 7 (D7) and day 28 (D28). **D:** Quantitative analysis of CD11b-positive area percentage. **E:** Quantitative analysis of IL-10-positive area percentage. Scale bars, 200 μm. **P* < 0.05, ***P* < 0.01, ****P* < 0.001, *****P* < 0.0001, ns: not significant.
